# Trimethylacetic Anhydride–Based Derivatization Facilitates Quantification of Histone Marks at the MS1 Level

**DOI:** 10.1016/j.mcpro.2021.100114

**Published:** 2021-06-12

**Authors:** Hana Kuchaříková, Pavlína Dobrovolná, Gabriela Lochmanová, Zbyněk Zdráhal

**Affiliations:** 1 Central European Institute of Technology, Masaryk University, Brno, Czech Republic; 2 Faculty of Science, Masaryk University, Brno, Czech Republic

**Keywords:** trimethylacetic anhydride, histone post-translational modifications, chemical derivatization, bottom–up proteomics, microwave irradiation, ACN, acetonitrile, DDA, data-dependent acquisition, DIA, data-independent acquisition, DMSO, dimethyl sulfoxide, EIC, extracted ion chromatogram, Enti, entinostat, HDACi, histone deacetylase inhibitor, hPTM, histone post-translational modification, Prop, propionic anhydride, PRM, parallel reaction monitoring, TFA, trifluoroacetic acid, TMA, trimethylacetic anhydride

## Abstract

Histone post-translational modifications (hPTMs) are epigenetic marks that strongly affect numerous processes, including cell cycling and protein interactions. They have been studied by both antibody- and MS-based methods for years, but the analyses are still challenging, mainly because of the diversity of histones and their modifications arising from high contents of reactive amine groups in their amino acid sequences. Here, we introduce use of trimethylacetic anhydride (TMA) as a new reagent for efficient histone derivatization, which is a requirement for bottom–up proteomic hPTM analysis. TMA can derivatize unmodified amine groups of lysine residues and amine groups generated at peptide N-termini by trypsin digestion. The derivatization is facilitated by microwave irradiation, which also reduces incubation times to minutes. We demonstrate that histone derivatization with TMA reliably provides high yields of fully derivatized peptides and thus is an effective alternative to conventional methods. TMA afforded more than 98% and 99% labeling efficiencies for histones H4 and H3, respectively, thereby enabling accurate quantification of peptide forms. Trimethylacetylation substantially improves chromatographic separation of peptide forms, which is essential for direct quantification based on signals extracted from MS1 data. For this purpose, software widely applied by the proteomics community can be used without additional computational development. Thorough comparison with widely applied propionylation highlights the advantages of TMA-based histone derivatization for monitoring hPTMs in biological samples.

## Highlights

• Microwave-assisted labeling of histones using TMA for bottom–up proteomics.
• TMA enables discrimination of isobaric peptides by improved chromatographic behavior.
• TMA facilitates histone quantification from MS1-level spectral information.
• TMA provides excellent performance for monitoring hPTM pattern in the tissues.

## Graphical abstract

**Figure:**
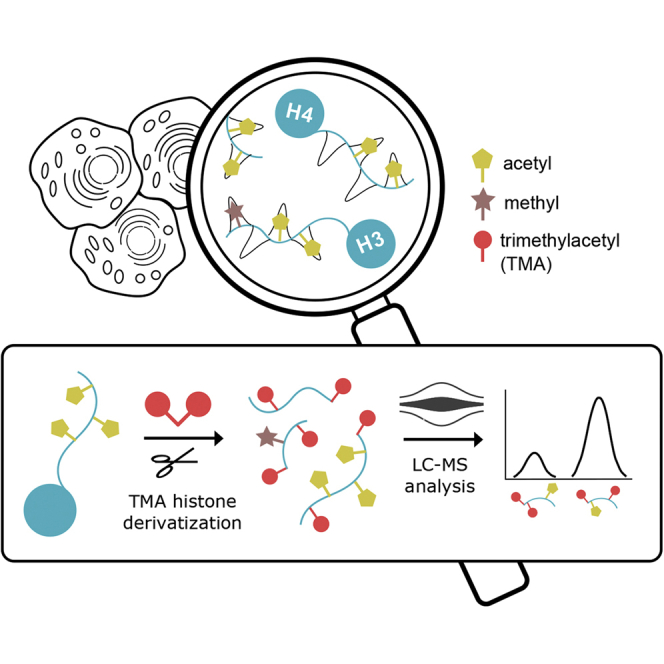

## In Brief

The discrimination of isobaric peptides represents a common challenge in histone characterization. This study reports TMA as a novel derivatization reagent for bottom–up proteomics of histone proteoforms. TMA substantially improves separation of positional isomers, which is a prerequisite for identification and quantification of post-translationally modified histone forms.

In recent years, scientists studying diverse biological phenomena have shown increasing interest in characterization of histone post-translational modifications (hPTMs), as they affect overall chromatin structure and form binding sites for effector molecules involved in numerous signaling pathways (1). Thus, together with DNA methylation and action of noncoding RNA, hPTMs are key factors in the regulation of processes that directly involve DNA, including gene expression, DNA repair, and replication. Hence, characterization of hPTMs is important for elucidation of fundamental regulatory principles in epigenetics and (*inter alia*) numerous clinical conditions (2). For example, aberrant regulation of hPTMs and recruitment of protein complexes is implicated in human diseases such as various autoimmune and neurological disorders and cancer, as recently reviewed (3, 4, 5).

Immunological techniques have been widely used to investigate hPTMs. Currently, chromatin immunoprecipitation coupled with next-generation sequencing is particularly valuable for mapping genomic regions linked to specific hPTMs. Two decades ago, MS emerged as a sensitive approach for identification and quantification of multiple PTM sites in specific histone variants. For example, using common proteomic pipelines including direct protein digestion with trypsin, the “zip model” hypothesis was confirmed, showing that acetylation of histone H4 proceeds in the Lys-16 to Lys-5 direction, whereas deacetylation proceeds in the reverse direction (6). By peptide mass fingerprinting using multiple proteases, more than 20 novel modification sites have been revealed that map not only to the globular core but also to the C-terminal tail domains of histones (7).

The quantitative capability of MS-based proteomics was clearly demonstrated in a functional analysis of histone deacetylase inhibitor (HDACi) PXD101 using LC–MS/MS. This resulted in quantification of 32 hPTMs at 29 sites from peptide ion intensities, including seven novel PTM sites in histones H2A, H2B, and H4 (8). Introduction of electron transfer dissociation/proton transfer reaction tandem MS enabled characterization of concurrently present PTMs on a single histone tail, thereby facilitating analysis of the roles of combinatorial histone modifications (9). Basically, all major proteomic approaches (top–down, middle–down, and bottom–up) and MS acquisition methods (data-dependent acquisition [DDA] and data-independent acquisition [DIA]) have been applied in studies of hPTMs, as recently reviewed (10, 11, 12). However, regardless of the method used, histone analysis is still challenging because of the extremely high number of histone variants and complex combinatorial patterns of their modifications. Growing evidence that histone epigenetic marks participate in various physiological and pathological processes has prompted further development of MS-based strategies for hPTM characterization (13, 14, 15). These include chemical derivatization of amine groups in histone sequences as a useful strategy for preparing samples for bottom–up LC–MS/MS analysis (16). A two-step derivatization process is usually applied, starting with NH_2_ labeling of the protein sequences to obtain longer and more hydrophobic Arg-C-type peptides after trypsin digestion, followed by labeling of newly released NH_2_ groups at peptide N-termini to increase the peptides' hydrophobicity and thus their chromatographic retention. Although various chemical agents (and conditions) for labeling have been tested, none have met all the requirements for appropriate and straightforward quantification of isobaric peptides at the MS1 level (11, 17, 18). For instance, use of propionic anhydride (Prop), the most well-established labeling agent for histones, does not enable separation of positional isomers of monoacetylated, diacetylated, and triacetylated peptides of H4 N-termini or certain diacetylated peptides of histone H3 on commonly used reversed stationary phases. In addition, there is a tendency for the so-called overpropionylation to occur, that is, side reactions at hydroxyl groups of serine, threonine, and tyrosine (17).

In the study presented here, we established a procedure for microwave-assisted labeling of histones prior to bottom–up LC–MS/MS analysis involving use of trimethylacetic anhydride (TMA; also known as pivalic anhydride) for lysine derivatization. This provided substantial advantages for histone quantification using MS1-level spectral information. Microwave irradiation enabled stepwise labeling of histone proteins and peptides, affording high derivatization efficiency. Here, we demonstrate the efficiency of TMA derivatization, chromatographic features of derivatized isobaric peptide forms, and variations in the mass spectrometric data. The derivatization protocol developed was successfully applied to distinguish hPTM states in human cell cultures treated with an HDACi and controls. For an impartial comparison of the TMA-based procedure, we also assessed the performance (using the same parameters) of the widely applied propionylation for labeling the same samples.

## Experimental Procedures

### Experimental Design and Statistical Rationale

The present study was designed with the following three aims. First, to establish a protocol for histone labeling using TMA. Second, to evaluate its performance and compare it to a well-established labeling technique (propionylation). Third, to investigate levels of TMA-labeled post-translationally modified histone peptides in cells grown under different conditions. For all measurements, histones extracted from the chronic lymphocytic leukemia cell line MEC-1 were used, and there were five biological replicates (*i.e.*, cultures in five separate flasks). Portions of each culture were treated with HDACi to alter histone modification status, whereas others were treated with dimethyl sulfoxide (DMSO) and used as control samples. Histone extracts were derivatized with either TMA or Prop. In total, 20 samples were subjected to LC–MS/MS analysis in DDA mode. To determine quantities of peptide forms in coeluting peaks, five TMA-labeled control samples were analyzed using MS in parallel reaction monitoring (PRM) mode. Raw data were searched in Mascot search engine through Proteome Discoverer 2.2 software (Thermo Fisher Scientific) with the settings described later; threshold ion score (Mascot search engine) for acceptable peptide identification was 30, and fragment match threshold for annotation was set to intensity of 10^4^. Identifications of selected histone peptides were manually verified and quantified based on peak areas derived from the extracted ion chromatograms (EICs) using Skyline software (version 19.1.1.248, MacCoss Lab Software), including identification alignment across the raw files based on retention time and *m/z* values. The significance of between-sample differences (control *versus* entinostat [enti]-treated, or TMA-*versus* Prop-labeled) was assessed by *t* tests. No outliers were detected in the datasets. Details of the statistical analyses are given later.

### Cell Cultivation and Treatment

The chronic lymphocytic leukemia cell line MEC-1 (DSMZ no.: ACC 497; German Collection of Microorganisms and Cell Cultures GmbH) was grown in Iscove's modified Dulbecco's medium (Gibco, Thermo Fisher Scientific) with phenol red, 10% (v/v) fetal bovine serum, 2 mM l-glutamine (Gibco, Thermo Fisher Scientific), and 100 IU penicillin/streptomycin at 37 °C and 5% CO_2_. Confluent cells were incubated with enti (MS-275; Cayman Chemicals) dissolved in DMSO, at a final concentration in the growth medium of 20 μM. Control cells were treated with a corresponding concentration of solvent, that is, 0.15% (v/v) DMSO. After 24 h of incubation, cells were collected and histones were extracted. Five replicates of each sample were prepared and analyzed.

### Histone Extract Preparation

Histone extracts were prepared following published procedures (20). Briefly, cells were washed twice in ice-cold PBS and incubated in lysis buffer consisting of 80 mM NaCl, 20 mM EDTA (Bio-Rad), 1% Triton X-100 (Carl Roth), 45 mM sodium butyrate, and 0.1 mM PMSF (Thermo Fisher Scientific) on ice for 20 min and centrifuged at 2000*g* for 8 min. Each resulting pellet was resuspended in 900 μl of ice-cold H_2_SO_4_ (Penta) and incubated at 4 °C for 2 h. Supernatant cleared by centrifugation at 16,000*g* and 4 °C for 8 min was diluted with 900 μl of 50% ice-cold trichloroacetic acid and incubated with shaking at 0 °C for 30 min. The resulting precipitate was harvested by centrifugation at 5000*g* at 4 °C for 30 min, washed with 50 mM HCl (Penta) in acetone and twice with acetone, then dried at room temperature. The prepared histone extract was dissolved in water, and the protein concentration of the solution was determined with the Bradford assay (using a Bio-Rad Kit).

### Histone Derivatization

Portions (12 μg) of histone extract were diluted to a final concentration of 1 μg μl^−1^ with 50% (v/v) acetonitrile (ACN; Honeywell). The pH was adjusted to 8 with NH_4_OH, and 3 μl of derivatization reagent consisting of TMA (Sigma–Aldrich) and ACN in a 1:3 (v/v) ratio was added to each sample. The samples were incubated for 5 h at room temperature with shaking, followed by repeated derivatization step including 16 h incubation, and then subjected to two rounds of microwave-assisted derivatization, as follows. Each sample was reduced in volume to approximately 5 μl in a microtube placed in a Savant SPD121P concentrator (Thermo Fisher Scientific), then diluted with 50% (v/v) ACN to a final volume of 12 μl. The microtube was capped and placed in a glass beaker that was covered with another glass beaker during incubation in a microwave oven for the first round of microwave-assisted derivatization. This consisted of three subcycles with the following steps: adjustment of the sample's pH to 8 with NH_4_OH, addition of 3 μl of derivatization reagent, and two 1-min incubations in the microwave oven at 350 W with a short centrifugation between them. The sample was then concentrated, and the second round of microwave-assisted derivatization was carried out with the same protocol. The resulting labeled protein sample was concentrated to 5 μl, and 0.3 μg of trypsin (sequencing grade modified; Promega Corporation) in 40 μl of 100 mM ammonium bicarbonate was added. After 4 h of incubation at 37 °C, another aliquot of 0.3 μg of trypsin was added, and the sample was incubated for a further 12 h at 37 °C. The generated peptides were then subjected to a round of microwave-assisted derivatization at N-termini using the protocol described previously, then the sample volume was reduced, 50% (v/v) ACN was added to a final volume of 24 μl, and another round of microwave-assisted derivatization was carried out with the same protocol. Labeled peptides were dried in a vacuum concentrator overnight. To ensure quantitative recovery of peptides, 60 μl of 50% ACN was added to each sample, followed by vacuum concentration to 15 μl. The sample was diluted with 100 μl of 0.1% trifluoroacetic acid (TFA) and desalted using a HyperSep SpinTip C18 (Thermo Fisher Scientific) following the manufacturer's instructions. Peptides were sequentially eluted with 20 μl of 0.1% TFA in 50% ACN and 2× 20 μl of 0.1% TFA in 75% ACN. Pooled eluates were transferred to an LC vial. Before LC–MS/MS, TFA was evaporated in a vacuum concentrator, and the sample was reconstituted in 0.1% formic acid (Honeywell). A step-by-step derivatization protocol including peptide purification is available in supplemental data (Section 1.1). Chemical derivatization using Prop was performed following a previously reported protocol (16) described in supplemental data (Section 2.1). All chemicals were purchased from Sigma–Aldrich unless otherwise specified.

### LC–MS/MS Analysis

Samples of both TMA- and prop-labeled peptides were spiked with iRT peptides (Biognosys) and analyzed using an Ultimate 3000 RSLCnano liquid chromatograph coupled to an Orbitrap Fusion Lumos Tribrid mass spectrometer (Thermo Fisher Scientific). Samples (2 μl) of peptide mixtures were injected, concentrated on an in-house trap column packed with X-Bridge BEH 130 C18 sorbent (100 μm × 30 mm, 3.5 μm particles; Waters), and separated on an Acclaim Pepmap100 C18 analytical column (75 μm × 500 mm, 3 μm particles; Thermo Fisher Scientific). Both columns were tempered at 40 °C. The mobile phases used for the gradient elution consisted of a binary mixture of 0.1% formic acid in water (A) and 0.1% formic acid in 80% ACN (B). Peptides were eluted with a 85 min gradient with a 300 nl.min^−1^ flow rate and content of B rising as follows: 5 to 25% (0–20 min), 25 to 29% (20–30 min), 29 to 32% (30–40 min), 32 to 38% (40–55 min), 38 to 50% (55–75 min), and 50 to 85% (75–85 min), followed by isocratic wash of 85% B (85–95 min). The outlet of the analytical column was connected to a Digital PicoView 550 ion source equipped with PicoTip SilicaTip emitter (New Objective) and Active Background Ion Reduction Device (ESI Source Solutions).

Mass spectra were acquired in data-dependent mode using 350 to 2000 *m/z* survey scans at a resolution of 60,000 (at *m/z* 200) with an automatic gain control target setting of 4 × 10^5^ and maximum injection time of 54 ms. Precursors with charge states from 2+ to 7+ and intensity above 1 × 10^4^ were subjected to higher energy collisional dissociation fragmentation with normalized collision energy of 30%. Once fragmented, precursors were excluded for 60 s before the next fragmentation. Precursors were isolated by quadrupole mass filtration with a 1.6 *m/z* isolation window. Tandem mass spectra were obtained for ions with *m/z* values of at least 110 using 30,000 resolution (at *m/z* 200). Ions were accumulated for a target value of 5 × 10^4^ or 500 ms injection time. The cycle time between master scans was 3 s. PRM data were acquired using survey scans with the same parameters as DDA. Tandem MS analysis was targeted to specific precursors (supplemental Table S1), which were selected in quadrupole with isolation window 1.6 *m/z*. The normalized higher energy collisional dissociation fragmentation energy was 30%. Fragment masses were monitored in the *m/z* range 110 to 2000 with 30,000 resolution (at *m/z* 200). Ions were accumulated for a target value of 5 × 10^4^ or 200 ms injection time.

### Data Analysis

Raw data acquired in DDA mode were searched against the modified cRAP contamination database (based on http://www.thegpm.org/crap/; 112 sequences), an in-house histone database (v150710; 114 protein sequences, generated from UniProt), and UniProt KB Human database (v180912; taxon ID: 9606; 21,053 sequences) using an in-house Mascot search engine (v2.6.2; Matrix Science) through Proteome Discoverer software (version 2.2.0.388). The mass error tolerances for precursors were 10 ppm for the cRAP database searches and 7 ppm for the others. Corresponding tolerances of MS/MS fragments were 0.5 and 0.03 Da, respectively. Enzyme specificity was set to semiArg-C with two missed cleavages allowed for all databases. Variable modifications set for individual databases were as follows: cRAP—acetylation (K), deamidation (N and Q), oxidation (M), trimethylacetylation (N-terminal region, K, S, T, and Y); Uniprot KB Human—acetylation (protein, N-terminal region, K) and trimethylacetylation (N-terminal region, K); in-house histone—the same modifications as for the Human database plus trimethylacetylation (S, T, and Y), methylation (K and R), dimethylation (K), and trimethylation (K). Search results were refined by applying Percolator with a 1% false discovery rate threshold to peptide spectrum matches. Identifications of selected peptides were manually inspected, and their quantity was determined and manually validated with Skyline software based on peak areas in EICs. Settings for database searches of propionylated peptides are described in supplemental data (Section 2).

### Statistical Analysis

To compare data acquired for TMA- and Prop-labeled samples in terms of reproducibility, the quantitative value of each peptide was log_10_ transformed. Means and SDs of the abundances of peptides detected and quantified following each derivatization technique were calculated and compared by Mann–Whitney tests.

The relative abundances of specific histone H4–modified peptide forms were calculated following a published approach (21). Briefly, areas of peaks assigned to all the forms were treated as composition. Data acquired from replicate samples were combined using geometric means and closure, then further transformed to calculate average compositions in the samples and hence relative abundances of the peptides (in log2 ratios). The KNIME Analytics Platform using R scripts was used for this analysis.

For quantitative comparison of peptide forms between control and enti-treated samples, log_10_-transformed values were normalized based on a maximal value of summed peptide peak areas in replicate arrays, the mean and SD were calculated for each peptide form, and the *t* test was applied. The fold difference in abundance between control and enti-treated samples was calculated for each peptide form, with the thresholds for statistical significance and fold change set to *p* < 0.01 and FC > 1.5, respectively.

**Fig. 5:**
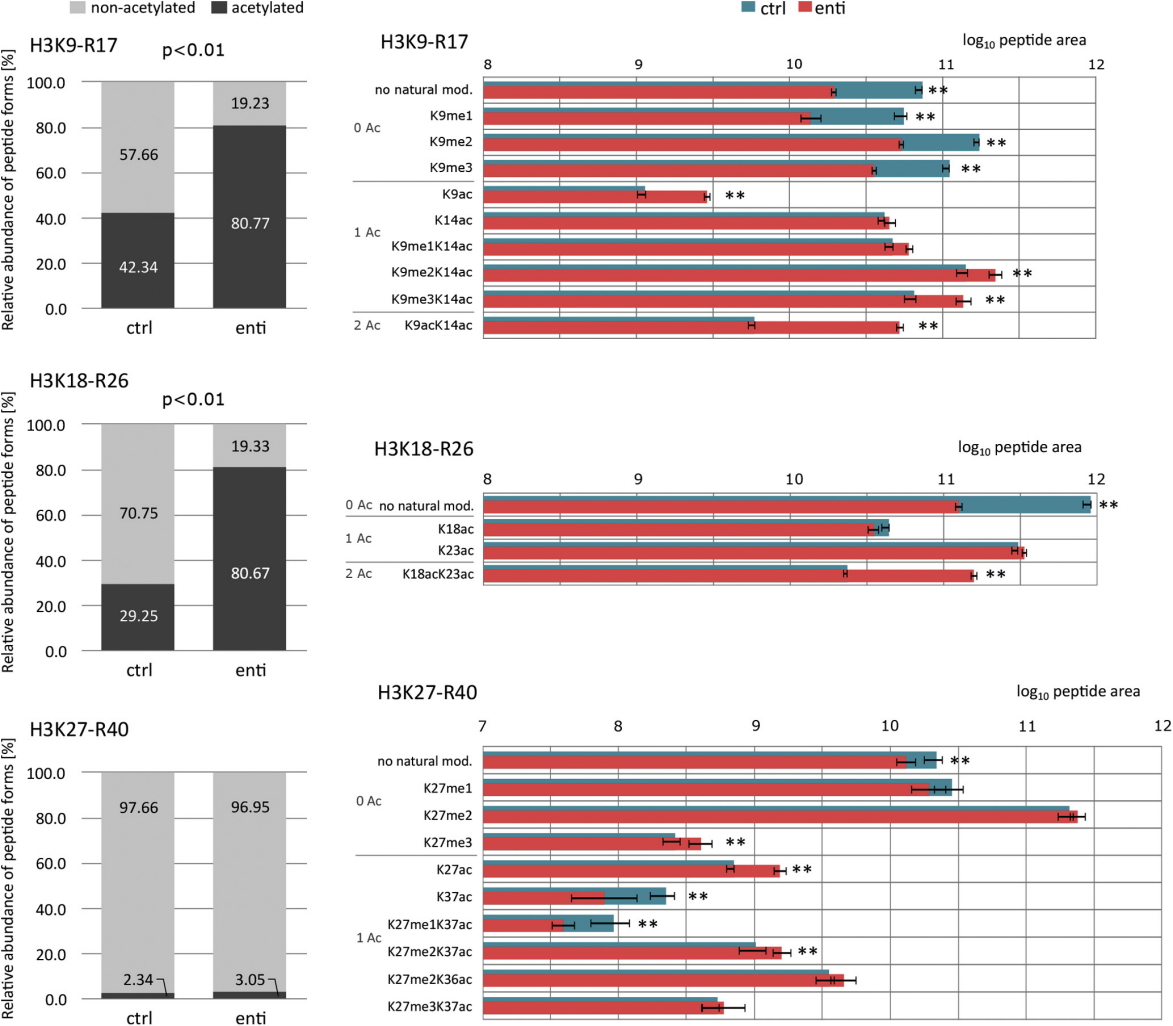
**Modification dynamics of histone H3 N-termini in enti-treated and control (ctrl) samples.** Five replicates of each group were subjected to LC–MS/MS analysis in DDA mode; mean values and SDs are presented. Relative proportions of nonacetylated and acetylated peptide forms (*left*) and the abundance of selected modified peptide forms after log_10_ transformation and normalization (*right*). *Asterisks* (∗∗) indicate significant differences (<0.01) between the groups, according to *t* tests, with >1.5 fold changes (supplemental Table S4). DDA, data-dependent acquisition; TMA, trimethylacetic anhydride.

**Fig. 6:**
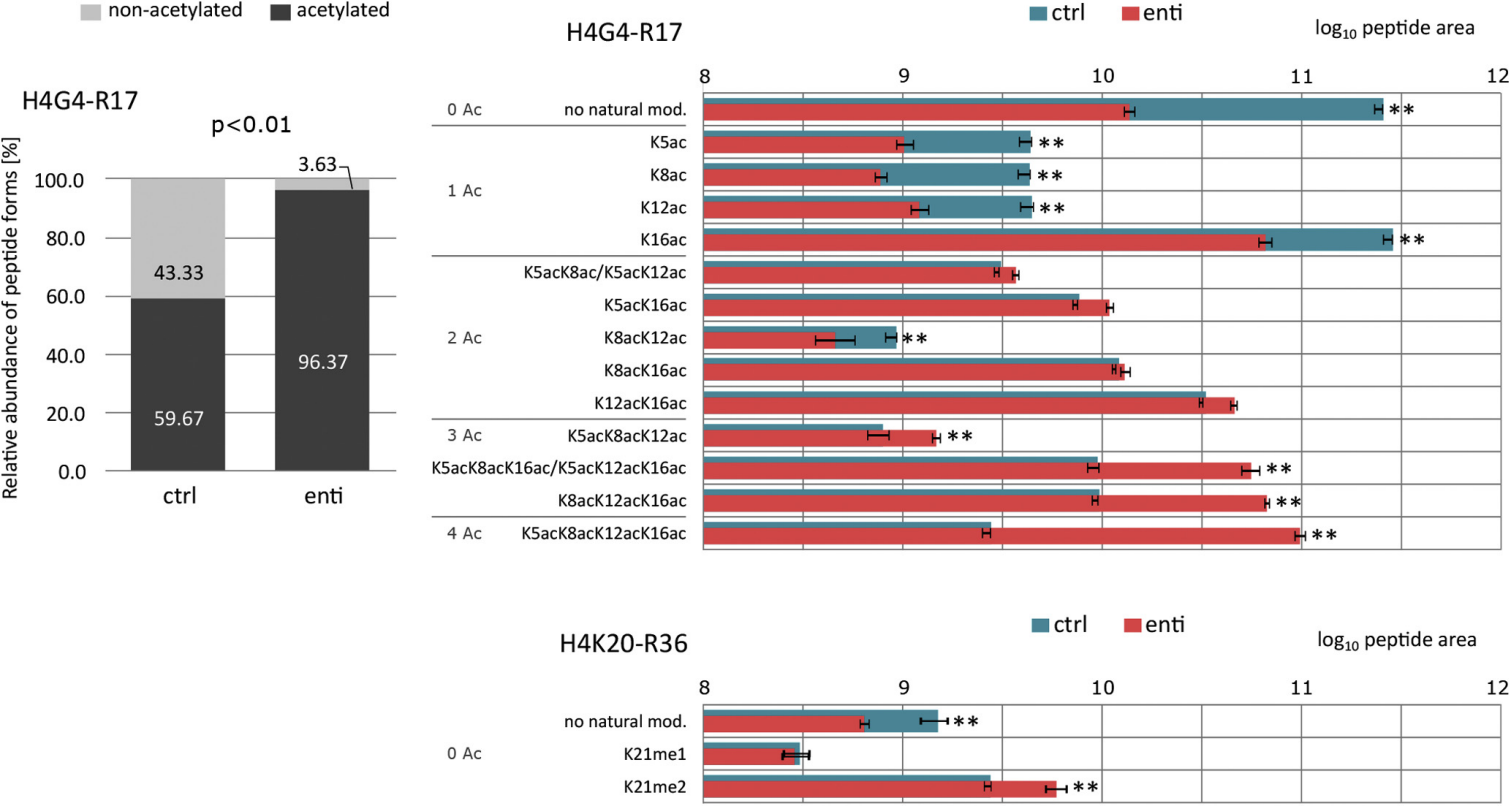
**Modification dynamics of histone H4 N-termini in enti-treated and control (ctrl) samples.** Five replicates of each group were subjected to LC–MS/MS analysis in DDA mode; mean values and SDs are presented. Relative proportions of nonacetylated to acetylated peptide forms of H4G4-R17 peptide (*left*) and the abundance of selected modified peptide forms after log_10_ transformation and normalization (*right*). Significant changes in methylation status were found in the H4K20-R36 peptide. *Asterisks* (∗∗) indicate significant differences (<0.01) between the groups, according to *t* tests, with >1.5 fold changes (supplemental Table S4). Coeluting H4G4-R17 peptide forms (K5acK8ac/K5acK12ac and K5acK8acK16ac/K5acK12acK16ac) can be distinguished and quantified based on PRM data as described in Experimental Procedures section. DDA, data-dependent acquisition; PRM, parallel reaction monitoring; TMA, trimethylacetic anhydride.

**Fig. 7:**
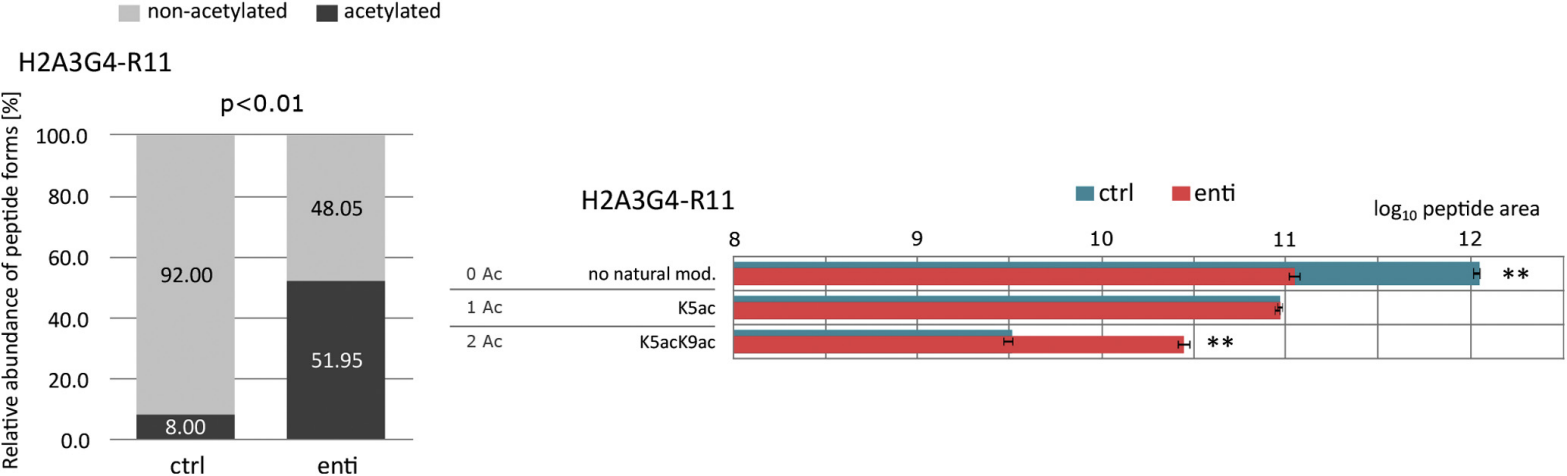
**Modification dynamics of histone H2A.3 N-termini in enti-treated and control (ctrl) samples.** Five replicates of each group were subjected to LC–MS/MS analysis in DDA mode; mean values and SDs are presented. Relative proportions of nonacetylated to acetylated peptide forms (*left*) and the abundance of selected modified peptide forms after log10 transformation and normalization (*right*). *Asterisks* (∗∗) indicate significant differences (<0.01) between the groups, according to *t* tests, with >1.5 fold changes (supplemental Table S4). DDA, data-dependent acquisition; TMA, trimethylacetic anhydride.

The overall acetylation status of enti-treated cells and corresponding controls was compared by calculating the abundance of all peptide forms containing acetyl group(s) relative to the abundance of all nonacetylated peptides. The resulting ratio was log2 transformed for each replicate separately, and the *t* test was used to assess the significance of differences in each specific form between control and enti-treated samples.

### Development and Analytical Validation of Targeted MS Measurements

PRM was used to determine quantities of peptide forms in coeluting peaks, specifically diacetylated and triacetylated forms of H4G4-R17 peptide. All five biological replicates of control samples labeled with TMA were analyzed. Precursor ions for PRM were selected based on the identifications in previous DDA. Their charge, *m/z*, and retention time window were used to create a list of parameters for targeted tandem MS analysis (supplemental Table S1). Quantities of diagnostic fragments for certain peptide forms were determined and manually validated using Skyline software based on peak areas in EICs (DIA; tier 3). The quantitative representation of coeluting isomers was calculated by assuming that the abundance (*A*) of coeluting isomers (*i*, *j*) is represented by their proportion (*R*) of the precursor peak area (*P*_*PA*_) in an MS1 EIC: $A_{i}=R_{i}\times P_{PA}$. For each isomer, *R* was calculated as the arithmetic mean of proportions of summed peak areas of isomer-specific y- and b-type fragments (*y*_*s*_, *b*_*s*_) (Equation 1).

(1) $$Ri=\frac{\frac{\sum y_{si}}{\sum y_{si}+\sum y_{sj}}+\frac{\sum b_{si}}{\sum b_{si}+\sum b_{sj}}}{2}$$

Equation 1 can be altered to calculate abundances of more coeluting peptides, if needed. If a precursor of a post-translationally modified histone peptide appeared in more than one charge state, abundances of isomers were calculated separately for each charge state and then summed.

## Results

### Workflow Description

**Fig. 1:**
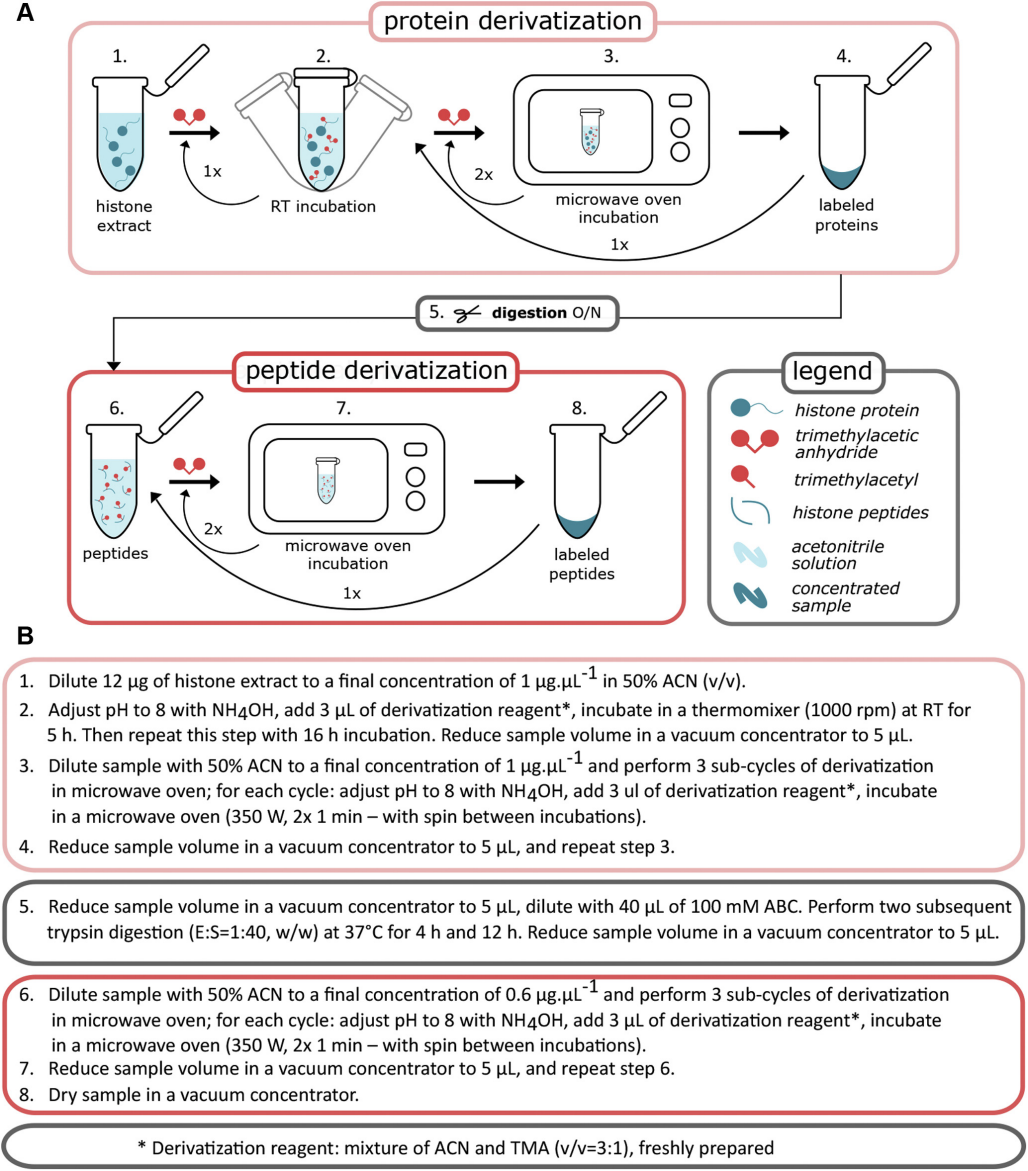
**Histone derivatization with TMA.***A*, illustrative scheme of the workflow. *B*, description of basic steps of the derivatization protocol. TMA, trimethylacetic anhydride.

The workflow for histone labeling using TMA consists of several sequential derivatization steps at both protein and peptide levels. In pilot experiments, we found that TMA labeling efficiency increased as incubation time was prolonged to 16 h. Subsequent inclusion of microwave-assisted incubation enabled efficient amine group derivatization with TMA within minutes. For the final version of the protocol presented here, we exploited the accelerated derivatization of amine groups by microwave irradiation while retaining overnight incubation for the first round of protein-level labeling. A scheme of the complete workflow is shown in Figure 1, and the protocol, including troubleshooting, is presented in detail in the supplemental data (Section 1). Sample preparation was optimized for 12 μg of histone extract, but obviously the amount of sample may be increased if the ratio between amounts of reagents and histone protein is maintained. Protocol performance (in terms of derivatization efficiency, chromatographic behavior of histone peptides, and relevance for detecting changes in histone modification profiles in real biological samples) was evaluated using mammalian histone extracts. In parallel, the same samples were also labeled with Prop to compare pros and cons of the two derivatization procedures (supplemental data, Section 2). Five biological replicates were used in all experiments, including TMA and Prop labeling.

**Fig. 2:**
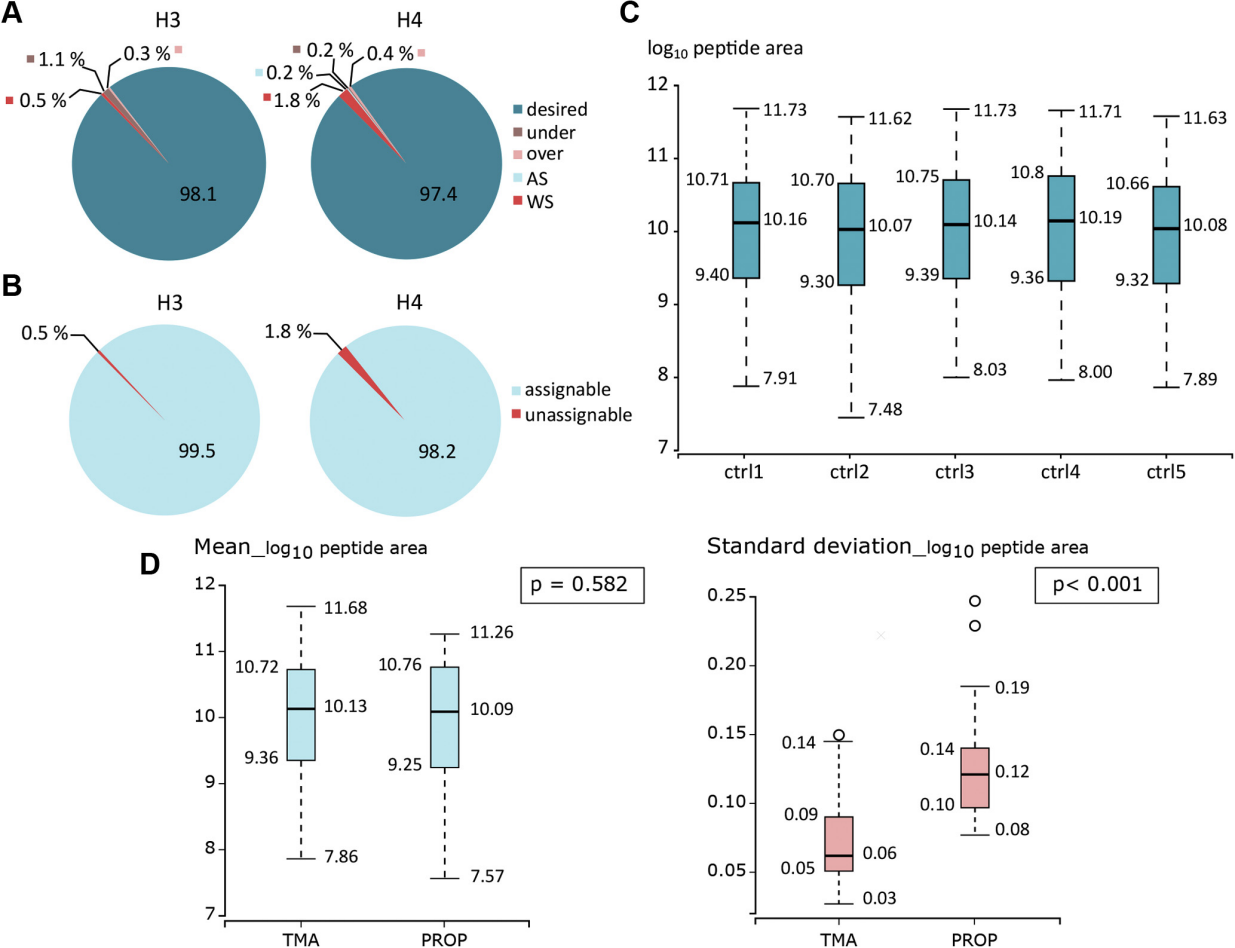
**Conversion rate and reproducibility of trimethylacetic anhydride (TMA) derivatization.***A*, proportions of histone H3 and H4 peptides in five categories: 1—*desired* (properly digested and fully derivatized), 2—*under* (properly digested but not completely derivatized), 3—*over* (properly digested and derivatized at S/T/Y residues), 4—*AS* (acceptable sequences, *i.e.*, peptides of various lengths with the same number of lysine residues as corresponding desired peptides), 5—*WS* (wrong sequences, *i.e.*, peptides with different numbers of lysine residue from desired peptides). *B*, proportions of assignable peptides, *i.e.*, peptides enabling correct quantification. *C*, distribution of log_10_-transformed precursor EIC peak areas of assignable peptides showing the reproducibility of histone derivatization across the replicates. The box plots show extremes, interquartile ranges, and medians (N = 41). *D*, comparison of means and SDs of histone H3 and H4 peptide forms' abundances in the TMA- and Prop-labeled samples. The box plots show extremes, interquartile ranges, and medians (N = 41 and 29 for TMA and Prop, respectively); *p* values were obtained from comparison of means and SDs by Mann–Whitney tests. EIC, extracted ion chromatogram.

**Fig. 3:**
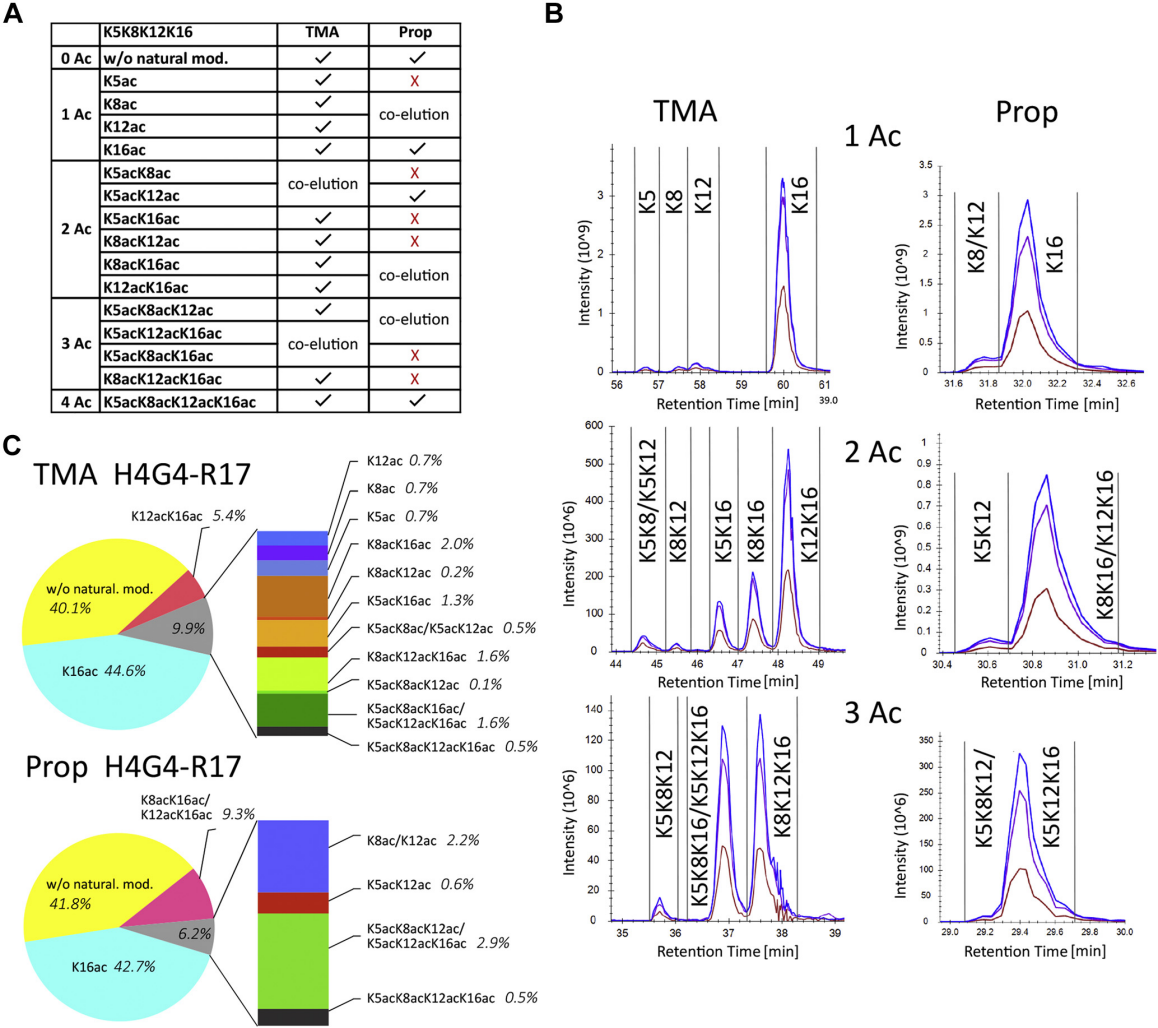
**Comparison of TMA- and Prop-derivatized H4G4-R17 peptide forms.***A*, overview of modified histone-peptide forms identified by LC–MS/MS. Peptide forms separated, using each derivatization agent, into individual peaks (✓), coeluting forms, and unidentified forms (x) are indicated. *B*, elution profiles of positional isomers of monoacetylated, diacetylated, and triacetylated peptides. *C*, representation of quantified forms. TMA, trimethylacetic anhydride.

**Fig. 4:**
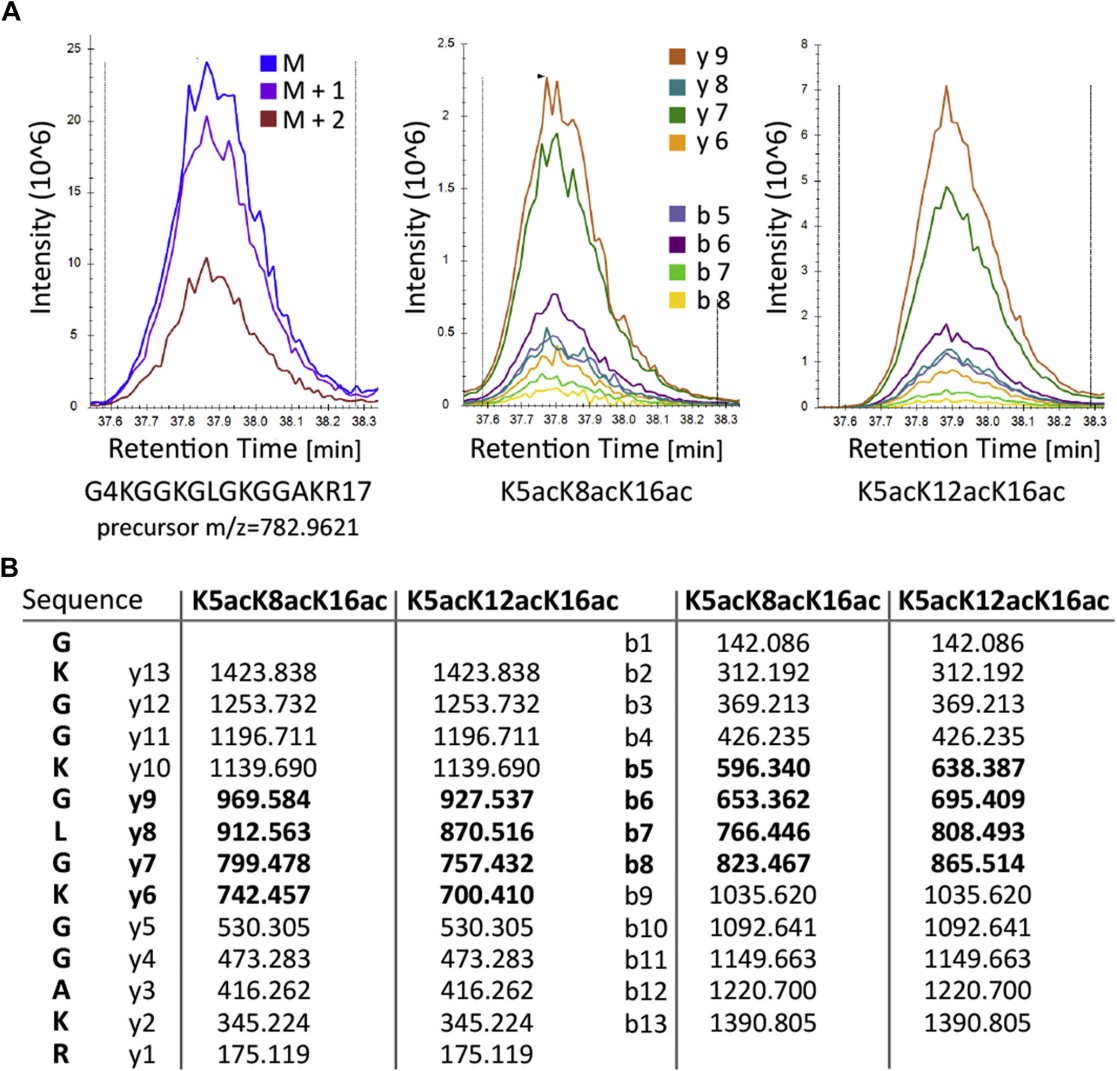
**Specific fragment ions of coeluting triacetylated forms of H4G4-R17 peptide after TMA derivatization.***A*, representative coeluting precursor peak of K5acK8acK16ac and K5acK12acK16ac forms and peaks of their y- and b-specific fragments obtained in LC–MS/MS PRM analysis. *B*, fragments of y- and b-ion series for K5acK8acK16ac and K5acK12acK16ac peptide forms with corresponding *m/z* values. Specific fragments for each form are marked in bold. PRM, parallel reaction monitoring; TMA, trimethylacetic anhydride.

### TMA Provided High Derivatization Efficiency of Histones

To assess derivatization efficiency and levels of accompanying unspecific reactions of the TMA labeling–based approach, we characterized histone peptide forms by LC–MS/MS. Selected post-translationally modified peptides of protein N-termini from both H3 and H4 histones were quantified using Skyline software. Evaluated peptide forms were classified into the following five subgroups to rate derivatization efficiency. First: *desired*—peptides cleaved at the C-terminus of arginine residues with complete labeling of all −NH_2_ groups. Second: *under*—peptides cleaved at the C-terminus of arginine with incomplete labeling (at least one −NH_2_ group remaining unmodified). Third: *over*—peptides cleaved at the C-terminus of arginine with unspecific labeling of −OH groups of serine (S), threonine (T), or tyrosine (Y), regardless of the degree of −NH_2_ labeling. Fourth: *acceptable sequence*—peptides of various lengths containing the same numbers of lysine residues as corresponding desired peptides, regardless of the degree of labeling. Fifth: *wrong sequence*—incorrectly cleaved peptides with different numbers of lysine residues from desired peptides (supplemental Table S2). The abundance of all evaluated peptides was summed, and calculated proportions of the subgroups show that more than 97% abundance of peptides of both histones H3 and H4 were in the desired category (Fig. 2*A* and supplemental data, Section 1.3). To ensure correct quantification of specific forms, peptides in subgroups 2 to 4 were assigned to corresponding desired peptides, collectively representing a higher-level category called “assignable.” The assignable category included more than 99% and 98% of the histone H3 and H4 peptides, respectively (Fig. 2*B* and supplemental data, Section 1.4), whereas the remaining wrong sequence peptides could not be correctly assigned to desired peptide forms or quantified and were thus called “unassignable.” The overall efficiency of TMA labeling, in terms of the percentage of assignable peptides, was comparable to results obtained following histone derivatization with Prop (supplemental data, Section 2.4). However, the proportion of desired histone H3 peptides was higher in TMA-labeled samples (~98%) than in Prop-labeled samples (~92%), mainly because of a lower frequency of unspecific labeling in TMA derivatization. The proportions of those undesired histone H3 peptides in the TMA- and Prop-labeled samples were 0.3% and 7.0%, respectively. Intersample variability of histone mark levels related to the preparation procedure prior to MS was evaluated using the same datasets. In total, 41 and 29 histone H3 and H4 assignable peptide forms identified in TMA- and Prop-labeled samples were selected for the evaluation. The variance in median values and interquartile ranges of log_10_-transformed peptide precursor areas across the replicates of TMA-labeled samples were <1.2% and <1.4%, respectively (Fig. 2*C* and supplemental data, Section 1.5), compared with <3.5% and <3.1% across propionylated samples (supplemental data, Section 2.4). The difference in mean log_10_-transformed peptide precursor areas between the two derivatization approaches was not significant (*p* = 0.582), but the SDs were significantly higher for Prop-labeled than TMA-labeled samples (*p* < 0.001; Fig. 2*D*).

### Mass Spectral Characteristics of TMA-Labeled Histone Peptides

Analogously to PTMs, chemical derivatization of amine groups results in production of low-mass signature fragment ions during tandem MS, including fragments corresponding to the derivatization label and natural PTMs at N-terminal lysines of peptides. Such ions can support unambiguous assignment of a given mass spectrum to a specific post-translationally modified peptide form. Inspection of MS/MS spectra of TMA-labeled histones revealed characteristic fragmentation peaks corresponding to masses of lysine immonium (*m/z* 101) and related ions (*m/z* 84 and 112) carrying TMA, including N-terminal lysine fragments carrying TMA together with methylation, dimethylation, and acetylation (supplemental data, Section 3.1), whereas no diagnostic peaks of lysine modified with TMA and trimethylation were detected in fragmentation spectra. Apparently, derivatization of monomethylated lysines is rarer when using TMA than in propionylation because of steric effects that block proton substitution.

### TMA Labeling Affords Better Chromatographic Separation of Histone Peptides Than Propionylation

Mammalian histone samples labeled with TMA were compared with those labeled with Prop in terms of number of identified post-translationally modified histone peptide forms and EIC profiles. The results show that TMA-labeled peptides had higher hydrophobicity, and their chromatographic peaks were more evenly distributed across the LC gradient than their propionylated counterparts (supplemental data, Section 3.2). Labeling of N-termini by TMA increased retention times by 3 to 6 min. Each TMA group increased the difference in retention time between TMA-labeled and Prop-labeled samples. Complete trimethylacetylation of naturally nonmodified peptide forms increased retention times by 17 to 42 min, depending on the number of lysine residues in the amino acid sequence (supplemental data, Section 3.2). The acquired hydrophobicity remarkably influenced the number of identified and quantified post-translationally modified peptide forms at MS1 level. Peptides selected for evaluation included modified forms of the following histone H3 and H4 N-terminal sequences: K9STGGKAPR17 (H3K9–R17), K18QLATKAAR26 (H3K18–R26), K27SAPATGGVKKPHR40 (H3K27–R40), and G4KGGKGLGKGGAKR17 (H4G4–R17). In total, 24 trimethylacetylated and 19 propionylated peptide forms of histones H3 were identified.

The advantages of TMA labeling were even more apparent for peptide sequences with multiple lysine residues (Fig. 3). Improved separation enabled identification of all 16 modified forms of H4G4-R17, whereas only ten forms were identified using propionylation because of missing MS/MS spectra of certain forms in coeluting peaks. Nonacetylated and tetra-acetylated forms were identified using both derivatization reagents (Fig. 3*A*). The final numbers of peptides quantified based on MS1 precursor ion intensities reflected the chromatographic behavior of peptides labeled with the two derivatization agents. In both cases, there was some coelution of positional isomers, but this issue was more problematic following propionylation. For instance, in total, 14 precursor peaks of H4G4-R17 peptide in TMA-labeled samples were quantified, compared with seven in Prop-labeled samples. In particular, all four isomers of the monoacetylated form were identified and separated in individual peaks, all six diacetylated isomers were identified in five peaks (as just one pair, K5acK8ac and K5acK12ac, coeluted), and all four isomers were identified within three peaks (because of coelution of K5acK8acK16ac and K5acK12acK16ac) in TMA-labeled samples. In contrast, following propionylation, only three forms of monoacetylated peptide were identified in two peaks (K16ac and coeluting K8ac/K12ac), three diacetylated forms were identified in two peaks (K5acK12ac and coeluting K8acK16ac/K12acK16ac), and two identified triacetylated isomers coeluted in a single peak (K5acK8acK12ac and K5acK12acK16ac; Fig. 3*B*). Clearly, the better chromatographic separation of the peptides after TMA derivatization enabled MS1 quantification of more forms than propionylation (Fig. 3*C*).

Quantities of some coeluting forms were determined based on the abundance of isomer-specific MS2 fragments. We obtained high-quality MS2 spectra of the precursors within a predefined retention time window by PRM. Specific fragments of both y- and b-ion series for each coeluting form were identified and quantified following the approach described in Experimental Procedures section. Proportions of isomers were calculated from precursor peak areas for both coeluting trimethylacetylated H4G4-R17 peptide pairs. Diacetylated K5acK8ac/K5acK12ac and triacetylated K5acK8acK16ac/K5acK12acK16ac positional isomers occurred in 1:5 and 1:3 ratios, respectively (supplemental Table S3). Results of a representative quantitative analysis showing precursor peaks of triacetylated positional isomers together with their specific fragments are displayed in Figure 4.

### Mass Spectra of TMA-Labeled Samples Reflect Dynamics of Histone Modification Status

The modification status of histones H3 and H4 was determined in cells treated with an HDACi, using TMA labeling followed by MS, to examine the procedure's ability to detect changes in histone modification in biological systems. Enti, HDACi with proven effectiveness in MEC-1 cells (20), was added to cultures of these cells at a final concentration of 20 μM, and its effect on histone acetylation status was examined after 24 h (see Experimental Procedures section for details).

A 38% increase in overall acetylation of H3K9-R17 peptide was detected, accompanied by a complex change in combinatorial pattern (Fig. 5). Levels of all histone marks except K14ac and K9me1K14ac forms differed significantly between enti-treated samples and controls. The higher acetylation state was accompanied by reduced levels of nonacetylated peptides, including those carrying a single group of me1 (methyl group), me2, or me3 at K9. The most pronounced increase in the global level of H3 peptides (51%) was in abundance of the fully acetylated H3K18-R26 form, whereas levels of monoacetylated peptides were not significantly affected by enti treatment. The level of global acetylation detected in the H3K27-R40 peptide was also similar in enti-treated and control samples, but detailed investigation revealed a significant increase in the K27ac form, accompanied by a lower level of the K37ac form. Moreover, enti-induced acetylation affected the abundance of forms carrying methylations at K27. The level of K27me1K37ac peptide was lower in enti samples than in control samples, whereas the abundance of the dimethylated counterpart and K27me3 form was significantly higher.

Although acquisition of higher hydrophobicity through TMA labeling enhanced peptides' retention in most cases, detection of H3A1-R8 peptide remained problematic. Only the nonmodified form of this peptide was identified in TMA-labeled samples, and methylated counterparts were lost. More details of this particular modified site can be found in supplemental data (Section 3.3). In addition to modified forms detected in the N-terminal part of the histone H3 sequence, the following modified sites were detected in its core or C-terminal tail: K56ac (Y54-R63), K79ac, K79me1, K79me2 (E73–R83), and K122ac (V117–R128). No significant difference (*p* < 0.05) in those peptide forms was detected between enti-treated samples and controls (supplemental Table S4).

However, a substantial (37%) increase in global acetylation of H4G4-R17 was detected (Fig. 6). The biggest quantitative differences between enti-treated and control samples were a more than 30-fold increase in abundance of the tetra-acetylated form and 17-fold decrease in the nonmodified form. Further, enti induced more than threefold decreases in abundance of all four monoacetylated isomers. Remarkably lower changes in levels of diacetylated isomers than in other forms were observed. Except for significant decreases in levels of the peptide acetylated at positions K8 and K12, the abundance of all other diacetylated forms did not differ or slightly increased. Triacetylated isomers with acetylation at K16 position substantially contributed to the hyperacetylation state in enti-treated samples (greater than sixfold change), as the K5acK8acK12ac peptide's abundance was twofold higher than in control samples. In addition, methylated R3 was identified in a peptide carrying multiple lysine acetylations (K8acK12acK16ac and K5acK8acK12acK16ac) in both control and enti-treated samples.

Enti treatment caused a significant increase in abundance of dimethylated H4K20-R36 peptide accompanied by a decrease in abundance of the nonmodified form (Fig. 6 and supplemental Table S4). In addition to the N-terminal region, lysine acetylations were detected at K31 (D24–R36), K59 (G56–R67), K77 (D68–R78), K79 and K91 (K79–R91) in histone H4 in TMA-treated samples. Levels of these peptide forms were not significantly affected by enti treatment (supplemental Table S4).

Although TMA derivatization was primarily optimized for characterization of histones H3 and H4, we checked its potential for detecting differences in PTM status of H2A and H2B between control and enti-treated samples. As the diversity of sequential variants of those histones within the chromatin is quite high, a representative variant of each of them—H2A type 3 (Q7L7L0) and H2B type 1-L (Q99880)—was used for detailed inspection of TMA-labeled post-translationally modified peptides. Besides cotranslational acetylation of S1 and methylation of R3, acetylations at positions K5 and K9 (G4–R11), K36 (K36–R42), and K95 (N89–R99) were detected in histone H2A. Importantly, enti treatment induced a significant increase in the level of diacetylated peptide G4–R11 and reduction in the level of its nonmodified counterpart (Fig. 7). For H2B, missing arginine residues in the N-terminal part of the sequence led to formation of long peptides with multiple lysines, *for example*, PELAKSAPAPKKGSKKAVTKAQKKDGKKR (P1–R29), which has 11 prospective sites (ten lysines plus the N-terminal amine group) of natural modification or chemical derivatization. Clearly, such long peptides are difficult to characterize using bottom–up approaches because of incomplete y-ion series and high combinatorial patterns of PTM sites. From manual inspection of MS/MS data, we were able to confirm the presence of acetylations at positions K5, K11, K12, K15, K16, and K20. However, the data did not enable determination of combinatorial patterns of multiple modified forms and quantification of their abundance.

Histone modification status was also examined in enti-treated and control samples labeled with Prop (supplemental data, Section 2.5 and supplemental Table S4). Although higher sequence coverage of histone proteins was obtained using Prop, a smaller number of modified peptide forms was identified and quantified. The overall acetylation trend was similar to that observed in TMA-treated samples, but detailed quantification of propionylated peptide forms was hindered by frequent presence of coeluting peaks of isobaric peptides in the MS1-level spectra and missing identification of certain modified sites (*e.g.*, H3K56ac, H3K79ac, H3K122ac, H4K31ac, H4K59ac, H4K77ac, H4K79ac, and H4K91ac). TMA enabled identification in majority of most acetylated and methylated forms annotated in the UniProt database or reported in previous studies (7, 8, 22). An overview of histone marks identified in amino acid sequences of histones H3.1, H4, and selected variants of H2A and H2B, including combinatorial patterns of the peptide forms, following TMA and Prop labeling is presented in supplemental data (Sections 3.4. and 3.5).

## Discussion

The altered properties of histone peptides because of derivatization of amine residues substantially affect MS-based quantification of modified forms. In addition to desired and advantageous changes, such as Arg-C–like digestion and improvements in peptide retention on chromatographic columns, chemical derivatization has several adverse effects that should be considered during data evaluation. The accurate quantification of histone marks in biological samples is hindered by unpredictable effects of peptide modifications (both added chemical labels and naturally occurring PTMs) on ionization efficiency (18, 23). However, relative values can be used to compare differences in histone mark levels associated with different biological conditions (24). Alternatively, previously published catalogs of LC–MS response factors, that is, correction factors determined by normalizing signals of acetylated and methylated synthetic peptides to the abundance of cleaved quantification tags, can be used to facilitate interpretation of histone marks' cellular abundance (23). The possibility that certain natural peptide forms may split into more chromatographic peaks because of side reactions or incomplete labeling should be also considered. Such drawbacks have been previously reported by several research groups for the widely used derivatization agent Prop (17, 18, 25, 26). During chemical derivatization of amino moieties, side products can be formed by reaction of acid anhydrides with hydroxyl groups of S, T, or Y. Although protocols including hydroxylamine treatment or boiling of propionylated peptides for the reversion of nonspecific O-acylation have been established (27), such approaches are not suitable for histone preparation as even natural PTMs are lost during hydrolysis of ester bonds. *N*-hydroxysuccinimide ester has reported potential as an amine-specific alternative to Prop, providing higher than 90% conversion rates of histone peptides (25). However, overpropionylation of certain peptides after N-hydroxysuccinimide ester labeling has also been described (17). Another disadvantage of propionylation is that it increases hydrophobicity less than derivatives obtained using various organic acid anhydrides (18). Moreover, propionylation does not satisfactorily improve chromatographic outcomes with conventionally used reversed stationary phases in terms of separation of positional isomers. Because of the presence of multiple lysines in the histone sequences, more than two isobaric peptides coelute quite frequently. The identification of peptides in coeluting peaks is hindered by intensity-based precursor selection in DDA mode. During peak elution, limited numbers of mass spectra can be obtained, and not all precursors in a coeluting peak will yield good fragmentation spectra that enable identification of the peptide forms.

The discrimination of isobaric peptides is a major challenge in quantification of post-translationally modified histone peptides. DIA is currently gaining increasing attention because of the possibility to integrate signals in EICs of both precursor and fragment ions, which reportedly leads to more accurate quantification (28, 29, 30, 31). However, this approach relies on a limited number of specific fragment ions in analyses of histone positional isomers.

The development of the procedure presented here was prompted by a need for better separation of specific modified histone peptide forms. In our experiments, evaluation of DDA datasets of TMA- and Prop-labeled histone peptides revealed comparable overall modification status in mammalian cell cultures. However, trimethylacetylation provided substantially better separation of positional isomers, thereby enabling direct quantification of most post-translationally modified peptide forms originating from histones H2A, H3, and H4 from MS1-level EICs using common proteomic software.

However, some particular histone peptides (*e.g.*, hydrophilic methylated H3K4 and long Arg-C–like N-terminal peptides of H2B variants) still pose challenges. For identifying methylated H3K4, Prop-PIC hybrid labeling seems to be the currently preferred method (26, 32, 33, 34, 35). This can reportedly shift retention times of monomethylated, dimethylated, and trimethylated forms of the T3-R8 peptide by 8 to 12 min (26). Such increases in hydrophobicity in combination with direct injection for LC and data acquisition based on a mass inclusion list for histone H3 could have substantially contributed to identification of the respective peptides. We presume that proton substitution with TMA at an N-terminal amino acid is probably not sufficient to improve retention of methylated T3-R8 peptides. Thus, further inspection and optimization of particular preparation steps of the presented protocol are needed, *that is*, purification of peptides on HyperSep SpinTip C18, their retention on trap columns, and identification using targeted data acquisition.

In addition, bottom–up proteomic approaches (with or without chemical derivatization of lysines) has limitations for characterizing PTMs of H2B histones. Because of sparse representation of arginine residues in sequences of H2B variants, multiple PTMs of long Arg-C–like peptides originating from derivatized H2B histones are difficult to localize. For instance, in this study, we identified acetylations at six lysine residues of P1-R29 peptide (K5, K11, K12, K15, K16, and K20) but could not determine their combinatorial pattern because of missing MS/MS fragment ions. On the other hand, trypsin digestion of H2B without derivatization generates multiple N-terminal peptides of different lengths, which also hinders quantitative analysis.

In summary, there is no universally suitable approach for PTM characterization of all core histones and their variants, and the analytical setup must be tailored in accordance with the main experimental objectives. However, the high efficiency and specificity of microwave oven–assisted TMA derivatization, together with its provision of highly reproducible quantitative data, clearly indicate that it is a reliable and effective alternative to established methods for preparing histone samples for bottom–up proteomic analysis. The presented method is ready to use for monitoring alterations in PTM patterns of histones H2A, H3, and H4 in cells, as demonstrated by our experiment with HDACi-treated human cell cultures. Most known acetylation and methylation sites were detected and quantified from MS1-level EICs, including isobaric peptides carrying multiple modifications. Thus, we anticipate that the TMA-based approach will facilitate investigation of epigenetic abnormalities associated with various developmental and pathological conditions, including human diseases.

## Data Availability

The MS proteomics data have been deposited to the ProteomeXchange Consortium *via* the PRIDE (19) partner repository with the dataset identifier PXD019502. The data-independent dataset has been deposited in the Panorama Public database and can be accessed *via* the URL: https://panoramaweb.org/Pi0wHl.url.

## Supplemental data

This article contains supplemental data (7, 8, 16, 22).

## Conflict of interest

The authors declare that the research was conducted in the absence of any commercial or financial relationships that could be construed as a potential conflict of interest. All authors have approved the final version of the article. All coauthors have contributed to the article and have seen and agreed with the contents of the article. The authors declare no financial interest to report. We certify that the submission is original work, has not been previously published, and has not been submitted for publication elsewhere while under consideration.
