# Supplementary material for: Trimethylacetic Anhydride–Based Derivatization Facilitates Quantification of Histone Marks at the MS1 Level
Source: Mol Cell Proteomics. 2021 Jun 12;20:100114. doi: 10.1016/j.mcpro.2021.100114 (PMC8283018; doi:10.1016/j.mcpro.2021.100114)
Supplement: Supplemental Data [file mmc5.docx]

Trimethylacetic anhydride-based derivatization facilitates quantification of histone marks at the MS1 level

Hana Kuchaříková^1,2^, Pavlína Dobrovolná^1^, Gabriela Lochmanová^1^* and Zbyněk Zdráhal^1,2^*

1 Central European Institute of Technology, Masaryk University, Kamenice 5, 625 00 Brno, Czech Republic

2 Faculty of Science, Masaryk University, Kamenice 5, 625 00 Brno, Czech Republic

*Authors for correspondence:

[gabriela.lochmanova@ceitec.muni.cz](mailto:gabriela.lochmanova@ceitec.muni.cz), zdrahal@sci.muni.cz

Running title: TMA for facilitated MS1-level quantification of histone marks

Table of contents

[1. Histone derivatization with TMA 3](#_Toc68689842)

[**1.1.** **Detailed protocol for TMA derivatization** 3](#_Toc68689843)

[**1.2.** **Troubleshooting** 5](#_Toc68689844)

[**1.3.** **Derivatization efficiency for histone H3 and H4** 5](#_Toc68689845)

[**1.4.** **Quantified peptides of histone H3 and H4** 6](#_Toc68689846)

[**1.5.** **Precursor peak areas reproducibility** 7](#_Toc68689847)

[2. Histone derivatization with Prop 8](#_Toc68689848)

[**2.1.** **Protocol for propionic anhydride derivatization** 8](#_Toc68689849)

[**2.2.** **LC-MS analysis** 8](#_Toc68689850)

[**2.3.** **Data analysis** 8](#_Toc68689851)

[**2.4.** **Propionic anhydride derivatization efficiency** 9](#_Toc68689852)

[**2.5.** **Propionic anhydride to monitor histone modification dynamics** 11](#_Toc68689853)

[3. Identification parameters of TMA- and Prop-labeled histone peptides 12](#_Toc68689854)

[**3.1.** **Mass spectral characteristics of TMA-labeled histone peptides** 12](#_Toc68689855)

[**3.2.** **Chromatographic behavior of TMA- and Prop-labeled histone peptides** 13](#_Toc68689856)

[**3.3.** **Identification of H3K4 in TMA-labeled samples** 16](#_Toc68689857)

[**3.4.** **Comparison of identified PTMs with the list of annotated PTMs in UniProt database** 17](#_Toc68689858)

[**3.5.** **Sequence coverage and PTMs identified in TMA- and Prop-labeled histone peptides** 19](#_Toc68689859)

[4. References 24](#_Toc68689860)

Supplemental Tables

Supplemental Table S1 (XLSX) - Precursors for PRM analysis

Supplemental Table S2 (XLSX) - Assigned and quantified peptide sequences

Supplemental Table S3 (XLSX) - Calculation of co-eluting forms’ quantities

Supplemental Table S4 (XLSX) - Data used to calculate abundance of histone modifications

# **Histone derivatization with TMA**

## **Detailed protocol for TMA derivatization**

**Material**

Acetonitrile (ACN, LC-MS grade, Honeywell, Czech Republic)

50% acetonitrile in HPLC grade water (v/v)

Trimethylacetic anhydride (99%, cat. No. 143502, Sigma Aldrich, MO, USA)

Ammonium hydroxide (28% NH_3_ in H_2_O, Sigma Aldrich)

Ammonium bicarbonate (Sigma Aldrich)

Trypsin (Sequencing grade modified, Promega Corporation, WI, USA)

*Note: Trimethylacetic anhydride is very reactive with the moisture in the air. Store the purchased stock solution under nitrogen or other inert gas blanket. You may prepare several aliquots (stored in amber glass vials) for separate derivatization rounds to reduce manipulation with stock solution and still work with fresh and reactive anhydride.*

1. Dilute 12 µg of histone extract with acetonitrile to a final protein concentration of 1 μg.μL^‑1^ in 50% acetonitrile (v/v).

*Note: Sample preparation was optimized for 12 μg, but the amount of the sample might be increased while maintaining a constant ratio of the reagents to histone protein amount.*

*Use fume hood for the subsequent steps where trimethylacetic anhydride is used.*

*The following part of the protocol (steps 2a, 3a, 6a) should be done in batches of maximum three samples.*

1. Derivatization at protein level – 1^st^ round…
2. Prepare derivatization reagent by mixing thrimethylacetic anhydride with acetonitrile in the ratio 1:3 (v/v); i.e., mix 3 μL trimethylacetic anhydride and 9 μL of acetonitrile, vortex and spin shortly down. Continue immediately without any interruption.
3. Add 0.5 μL of ammonium hydroxide to the sample, and immediately add 3 μL of freshly prepared derivatization reagent. Add 1 μL of ammonium hydroxide, check pH using a pH indicator paper strip and adjust to 8 with additional ammonium hydroxide.
4. Incubate sample in thermomixer with 1000 rpm at RT for 5 h.

Repeat steps 2a, b and incubate in thermomixer with 1000 rpm at RT for 16 h (overnight).

1. Reduce the sample volume to approximately 5 μL in vacuum concentrator, add 50% acetonitrile to a final protein concentration of 1 μg.μL^-1^.

*Note: The vacuum concentration is used to reduce content of unreacted reagents.*

1. Derivatization at protein level – 2^nd^ round…
2. Add 0.5 μL of ammonium hydroxide to the sample, and immediately add 3 μL of freshly prepared derivatization reagent (described in 2a). Add 1 μL of ammonium hydroxide, check pH using a pH indicator paper strip and adjust to 8 with additional ammonium hydroxide. Incubate in microwave oven at 350 W twice for one minute, spin in between.

*Note:* *Always use glass beakers to hold and cover microtubes in microwave oven, change beakers when they are hot.*

*Recommended microwave oven power output is 350 W, however, the most suitable value should be tested since each device may produce different effective power output, especially when using older machines.*

1. Repeat step 3a twice more (3 sub-cycles of derivatization in total), cool the samples at RT in between.
2. Reduce the sample volume to approximately 5 μL in vacuum concentrator, add 50% acetonitrile to a final protein concetration of 1 μg.μL^-1^, and repeat the derivatization with incubation in microwave oven (3x step 3a) to ensure high efficiency of the labeling.
3. Protein digestion…
4. Reduce the sample volume to approximately 5 μL in vacuum concentrator. Dilute the sample with 100 mM ammonium bicarbonate to a final concentration of 0.3 μg.μL^‑1^.

Perform two subsequent trypsin digestions (E:S=1:40, w/w, each) at 37°C with incubation for 4 h and 12 h, respectively.

*Note: Inspect the sample for a precipitate presence after 30 minutes of incubation. If the precipitate appears, add ¼ of total volume of 100% acetonitrile.*

1. Reduce the sample volume to approximately 5 μL in vacuum concentrator. Dilute sample with 50% acetonitrile to a final peptide concentration of 0.6 μg.μL^-1^.
2. Derivatization at peptide level – 1^st^ round …
3. Add 0.5 μL of ammonium hydroxide to the sample, and immediately add 3 μL of freshly prepared derivatization reagent (described in 2a). Add 1 μL of ammonium hydroxide, check pH using a pH indicator paper strip and adjust to 8. Incubate in microwave oven at 350 W twice for one minute, spin in between.
4. Repeat step 5a twice more (3 sub-cycles of derivatization in total).
5. Derivatization at peptide level – 2^nd^ round

Reduce the sample volume to approximately 5 μL in vacuum concentrator. Dilute sample with 50% acetonitrile to a final peptide concentration of 0.6 μg.μL^-1^ and repeat the derivatization with incubation in microwave oven (3x step 5a).

1. Dry out the sample in vacuum concentrator overnight.

Before LC-MS analysis perform sample desalting, e.g. with HyperSep SpinTip C18 (Thermo Fisher Scientific) according to following protocol.

Reconstitute sample in 60 μL of 50% ACN and concentrate to 15 μL, dilute with 100 μL 0.1% TFA (trifluoroacetic acid), check that pH is below 3 with pH indicator paper. Rehydrate the SpinTip with 4×20 μL 0.1% TFA in 50% ACN, then equilibrate with 5×20 μL 0.1% TFA. Load sample sequentially. Wash the SpinTip with 5×20 μL 0.1% TFA. Elute the sample sequentially with 20 μL 0.1% TFA in 50% ACN and 2×20 μL 0.1% TFA in 75% ACN. Quantitatively transfer sample to LC vial, concentrate to reduce TFA content and dissolve in 0.1% formic acid.

## **Troubleshooting**

| **Issue** | **Solution suggestion** |
| --- | --- |
| Vial is overheating in microwave oven | Use more concentrated histone extract to reduce the sample volume during derivatization. Test the optimal microwave oven power output. |
| White precipitate appears in vial during digestion. | Add ¼ of total volume of 100% ACN. |
| Sample was completely evaporated during vacuum concentration. | No significant sample losses occur. Dissolve sample in 2/3 of final volume of 50% ACN, tapping the vial bottom by finger for 30 s, if not dissolved add the rest volume of 100% ACN, vortex. |
| Derivatization efficiency suddenly decreased and WS peptides became more abundant  (WS - Wrong Sequence; peptides containing different number of lysine residues than peptides cleaved only at C-terminus of arginine). | Use fresh trimethylacetic anhydride reagent. Make sure you store it properly. |

## **Derivatization efficiency for histone H3 and H4**

Peptide abundance in individual categories (desired, under, over, acceptable sequence (AS), and wrong sequence (WS)) for each biological replicate presented as precursor peak areas in MS1 EIC (narrower tables) and as percentage of each group from corresponding summed area (wider tables). Wider tables contain a mean value (represented in pie chart in Figure 2A) and standard deviation.

| H3 | ctrl 1 | ctrl 2 | ctrl 3 | ctrl 4 | ctrl 5 |  |  |
| --- | --- | --- | --- | --- | --- | --- | --- |
| desired | 1.63E+12 | 1.24E+12 | 1.63E+12 | 1.66E+12 | 1.28E+12 |  |  |
| under | 1.51E+10 | 2.83E+10 | 1.75E+10 | 7.89E+09 | 1.01E+10 |  |  |
| over | 3.62E+09 | 3.66E+09 | 4.82E+09 | 5.37E+09 | 4.44E+09 |  |  |
| AS | 0.00E+00 | 0.00E+00 | 0.00E+00 | 0.00E+00 | 0.00E+00 |  |  |
| WS | 9.25E+09 | 6.02E+09 | 9.60E+09 | 8.37E+09 | 7.16E+09 |  |  |
| summed area | 1.65E+12 | 1.28E+12 | 1.67E+12 | 1.68E+12 | 1.30E+12 |  |  |
|  |  |  |  |  |  |  |  |
| H3 | ctrl 1 | ctrl 2 | ctrl 3 | ctrl 4 | ctrl 5 | average | ST DEV |
| desired | 98.31% | 97.04% | 98.09% | 98.71% | 98.33% | 98.09% | 0.63% |
| under | 0.92% | 2.21% | 1.05% | 0.47% | 0.78% | 1.08% | 0.66% |
| over | 0.22% | 0.29% | 0.29% | 0.32% | 0.34% | 0.29% | 0.05% |
| AS | 0.00% | 0.00% | 0.00% | 0.00% | 0.00% | 0.00% | 0.00% |
| WS | 0.56% | 0.47% | 0.58% | 0.50% | 0.55% | 0.53% | 0.04% |
| sum | 100.00% | 100.00% | 100.00% | 100.00% | 100.00% |  |  |

| H4 | ctrl 1 | ctrl 2 | ctrl 3 | ctrl 4 | ctrl 5 |  |  |
| --- | --- | --- | --- | --- | --- | --- | --- |
| desired | 5.35E+11 | 3.81E+11 | 5.17E+11 | 4.61E+11 | 4.13E+11 |  |  |
| under | 2.25E+09 | 2.13E+09 | 3.26E+09 | 9.59E+08 | 1.73E+09 |  |  |
| over | 1.01E+09 | 5.13E+08 | 1.07E+09 | 1.29E+09 | 1.06E+09 |  |  |
| AS | 1.36E+09 | 7.94E+08 | 1.17E+09 | 5.69E+08 | 4.79E+08 |  |  |
| WS | 1.35E+10 | 6.93E+09 | 1.13E+10 | 6.26E+09 | 5.03E+09 |  |  |
| summed area | 5.53E+11 | 3.91E+11 | 5.34E+11 | 4.70E+11 | 4.22E+11 |  |  |
|  |  |  |  |  |  |  |  |
| H4 | ctrl 1 | ctrl 2 | ctrl 3 | ctrl 4 | ctrl 5 | MEAN | ST DEV |
| desired | 96.73% | 97.35% | 96.86% | 98.07% | 98.03% | 97.4% | 0.6% |
| under | 0.41% | 0.54% | 0.61% | 0.20% | 0.41% | 0.4% | 0.2% |
| over | 0.18% | 0.13% | 0.20% | 0.28% | 0.25% | 0.2% | 0.1% |
| AS | 0.25% | 0.20% | 0.22% | 0.12% | 0.11% | 0.2% | 0.1% |
| WS | 2.43% | 1.77% | 2.12% | 1.33% | 1.19% | 1.8% | 0.5% |

## **Quantified peptides of histone H3 and H4**

Abundance of assignable and unassignable peptide forms of H3 and H4 proteins in each biological replicate presented as summed peaks area in MS1 EIC of respective peptides (narrower table), and as percentage from corresponding summed area (wider table).

| H3 | ctrl 1 | ctrl 2 | ctrl 3 | ctrl 4 | ctrl 5 |  |  |
| --- | --- | --- | --- | --- | --- | --- | --- |
| assignable | 1.65E+12 | 1.28E+12 | 1.66E+12 | 1.67E+12 | 1.29E+12 |  |  |
| unassingnable | 9.25E+09 | 6.02E+09 | 9.60E+09 | 8.37E+09 | 7.16E+09 |  |  |
| summed area | 1.65E+12 | 1.28E+12 | 1.67E+12 | 1.68E+12 | 1.30E+12 |  |  |
|  |  |  |  |  |  |  |  |
| H3 | ctrl 1 | ctrl 2 | ctrl 3 | ctrl 4 | ctrl 5 | MEAN | ST DEV |
| assignable | 99.44% | 99.53% | 99.42% | 99.50% | 99.45% | 99.47% | 0.04% |
| unassingnable | 0.56% | 0.47% | 0.58% | 0.50% | 0.55% | 0.53% | 0.04% |
| sum | 100.00% | 100.00% | 100.00% | 100.00% | 100.00% |  |  |

| H4 | ctrl 1 | ctrl 2 | ctrl 3 | ctrl 4 | ctrl 5 |  |  |
| --- | --- | --- | --- | --- | --- | --- | --- |
| assignable | 5.40E+11 | 3.84E+11 | 5.23E+11 | 4.64E+11 | 4.17E+11 |  |  |
| unassingnable | 1.35E+10 | 6.93E+09 | 1.13E+10 | 6.26E+09 | 5.03E+09 |  |  |
| summed area | 5.53E+11 | 3.91E+11 | 5.34E+11 | 4.70E+11 | 4.22E+11 |  |  |
|  |  |  |  |  |  |  |  |
| H4 | ctrl 1 | ctrl 2 | ctrl 3 | ctrl 4 | ctrl 5 | MEAN | ST DEV |
| assignable | 97.57% | 98.23% | 97.88% | 98.67% | 98.81% | 98.2% | 0.5% |
| unassingnable | 2.43% | 1.77% | 2.12% | 1.33% | 1.19% | 1.8% | 0.5% |

## **Precursor peak areas reproducibility**

Areas of assignable peptides were log_10_-transformed and their distribution is displayed in box-plot (Figure 2C, N = 41). Following tables describe the data presented in box-plot and their reproducibility.

|  | TMA_ctrl 1 | TMA_ctrl 2 | TMA_ctrl 3 | TMA_ctrl 4 | TMA_ctrl 5 |
| --- | --- | --- | --- | --- | --- |
| min | 7.91 | 7.48 | 8.03 | 8.00 | 7.89 |
| max | 11.73 | 11.62 | 11.73 | 11.71 | 11.63 |
| median | 10.16 | 10.07 | 10.14 | 10.19 | 10.08 |
| 1st quar | 9.40 | 9.30 | 9.39 | 9.36 | 9.32 |
| 3rd quar | 10.71 | 10.70 | 10.75 | 10.80 | 10.66 |

|  | max | min | difference (max-min) | diff./max | diff./min |
| --- | --- | --- | --- | --- | --- |
| median | 10.19 | 10.07 | 0.12 | 1.18% | 1.19% |
| 1st quar | 9.40 | 9.30 | 0.10 | 1.06% | 1.08% |
| 3rd quar | 10.8 | 10.66 | 0.14 | 1.30% | 1.31% |

# **Histone derivatization with Prop**

## **Protocol for propionic anhydride derivatization**

Histones extracted from MEC-1 cells were subjected to a double round of propionic anhydride derivatization (at both protein and peptide levels) based on previously published work (16) with modifications. Briefly, a 12 μg portion of histone sample was diluted with acetonitrile (ACN) and deionized water to a final volume of 12 μL and final ACN concentration of 50 % (v/v). NH_4_OH (0.5 μL) was added, then propionylation reagent was prepared by mixing propionic anhydride with ACN in a 1:3 ratio and a portion equal to 25 % of the sample volume was immediately added. The pH was adjusted to 8–9 by NH_4_OH, the sample was incubated in thermomixer at 37 °C and 700 rpm for 20 min, then the sample volume was reduced in a Savant SPD121P concentrator (SpeedVac; Thermo Scientific) to 5 μL. The second round of propionylation was carried out with the same protocol. Propionylated histone proteins were reconstituted in 12 μL of 100 mM ammonium bicarbonate (AB) and trypsin was added in a 1:40 enzyme:protein ratio. After overnight digestion at 37 °C, the sample was dried in the SpeedVac. The generated peptides were subjected to a double round of propionylation at N-termini using the protocol described above. Sample was diluted with 100 μL of 0.1% trifluoracetic acid (TFA), desalted on HyperSep SpinTip C18 according to manufacturer instructions. Sequential elution of peptides was performed using 20 μL of 0.1% TFA in 50% ACN and 2x 20 μL of 0.1% TFA in 75% ACN, respectively. Pooled eluates were transferred to LC vial. Prior to LC-MS/MS, TFA was evaporated in vacuum concentrator and the sample was reconstituted in 0.1% formic acid.

## **LC-MS analysis**

The LC-MS analysis of Prop-derivatized samples was performed the same way as for TMA-derivatized samples. Detail information about the analysis is provided in the main text in section Material and Methods.

## **Data analysis**

Raw data were searched against the modified cRAP contamination database (based on http://www.thegpm.org/crap/, 112 sequences), in-house histone database (v150710, 114 protein sequences), and UniProt KB Human database (v180912, taxon ID: 9606, 21053 sequences) using in-house Mascot search engine (v2.6.2, Matrix Science, United Kingdom) through Proteome Dicoverer software (v2.2.0.388, Thermo Fisher Scientific). Mass error tolerance was 7 ppm (10 ppm for cRAP) and 0.03 Da (0.5 Da for cRAP) for precursors and MS2 fragments, respectively. Enzyme specificity was set to semiArg-C with 2 missed cleavages allowed for all databases. Variable modifications were as follows for individual databases: cRAP ‑ acetylation (K), deamidation (N, Q), oxidation (M), propionylation (N-term, K, S, T, Y); Uniprot KB Human ‑ acetylation (protein N-term, K) and propionylation (N-term, K), in house histone – the same modifications as for Human database plus propionylation (S, T, Y), methylation (K, R), dimethylation (K), trimethylation (K). Search results were refined at 1 % false discovery rate with Percolator node on peptide spectrum match level. Selected peptide identifications were manually inspected, their quantity was determined and manually validated in Skyline software (v19.1.1.248) based on peak areas in EICs (extracted ion chromatograms).

## **Propionic anhydride derivatization efficiency**

The conversion rate of propionic anhydride derivatization. (section A) Proportions of histone H3 and H4 peptides in five categories: 1 – desired (properly digested and fully derivatized), 2 ‑under (properly digested, but not completely derivatized), 3 – over (properly digested and derivatized on S/T/Y residue), 4 – AS (Acceptable Sequence; shorter or longer peptides containing the same number of lysine residues as corresponding desired peptides, 5 – WS (Wrong Sequence; peptides containing different number of lysine residues than desired peptides). (section B) The proportion of assignable peptides, i.e., peptides enabling correct quantification. (section C) Distribution of log_10_-transformed precursor EIC peak areas of assignable peptides showing the reproducibility of histone derivatization. The box-plots show extremes, interquartile ranges and medians (N = 29).


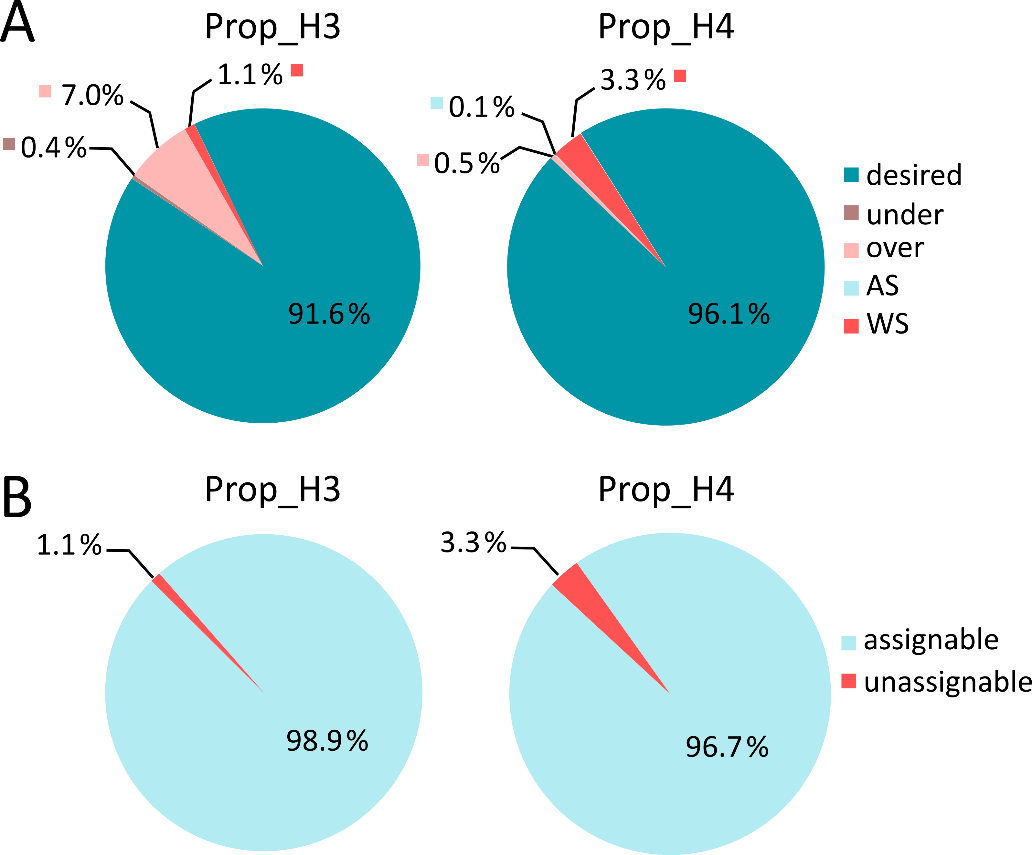


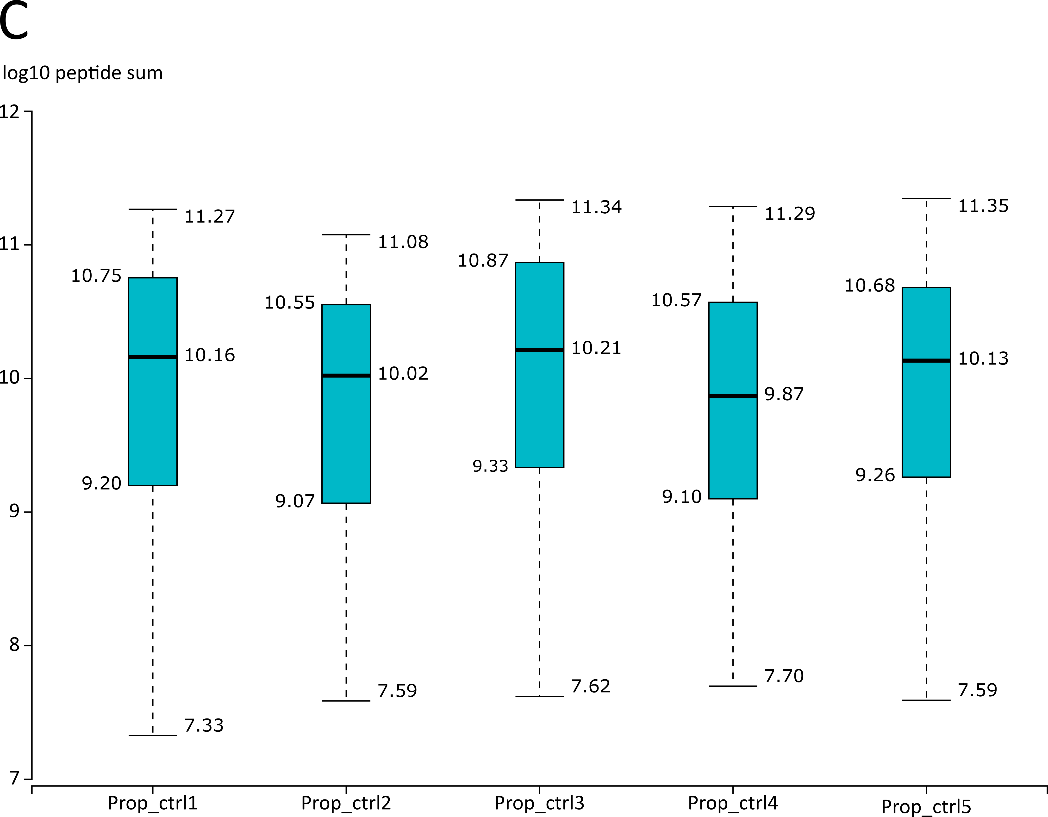


Following tables describe data presented in box-plots and their reproducibility.

|  | Prop_ctrl 1 | Prop_ctrl 2 | Prop_ctrl 3 | Prop_ctrl 4 | Prop_ctrl 5 |
| --- | --- | --- | --- | --- | --- |
| min | 7.33 | 7.59 | 7.62 | 7.70 | 7.59 |
| max | 11.27 | 11.08 | 11.34 | 11.29 | 11.35 |
| median | 10.16 | 10.02 | 10.21 | 9.87 | 10.13 |
| 1st quar | 9.20 | 9.07 | 9.33 | 9.10 | 9.26 |
| 3rd quar | 10.75 | 10.55 | 10.87 | 10.57 | 10.68 |

|  | max | min | difference (max-min) | diff./max | diff./min |
| --- | --- | --- | --- | --- | --- |
| median | 10.21 | 9.87 | 0.34 | 3.33% | 3.44% |
| 1st quar | 9.33 | 9.07 | 0.26 | 2.79% | 2.87% |
| 3rd quar | 10.87 | 10.55 | 0.32 | 2.94% | 3.03% |

## **Propionic anhydride to monitor histone modification dynamics**

Modification dynamics of histone H3, H4 and H2A N-termini induced by enti-treatment. Five replicates of each group were carried out and mean values are presented. The relative proportion of non-acetylated to acetylated peptide forms (left), and the abundance of selected modified peptide forms after log10 transformation and normalization (right). T-test determined the statistical significance between both groups, threshold set at **p<0.01, and fold‑change > 1.5 (Supplemental Table S4).


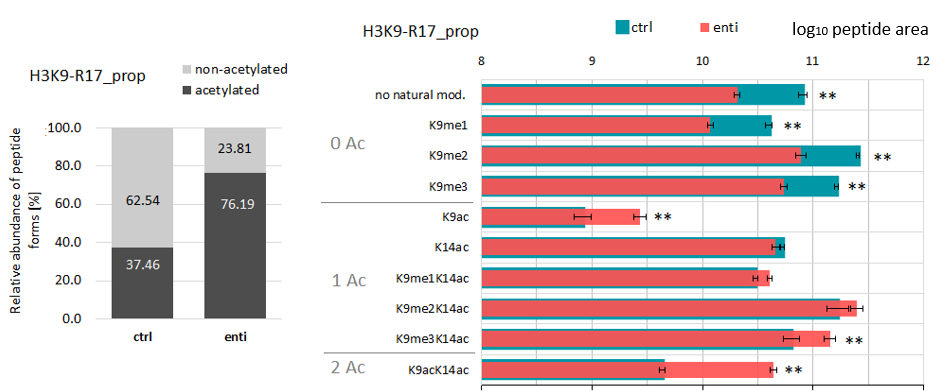


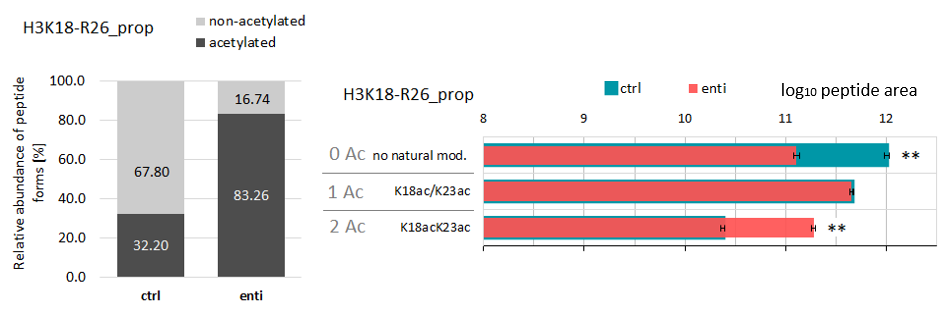


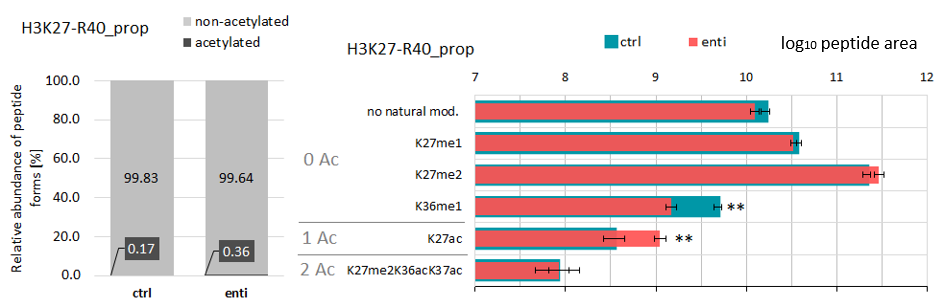


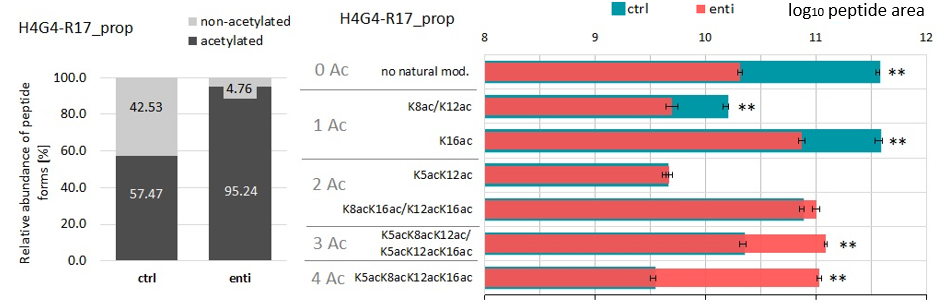


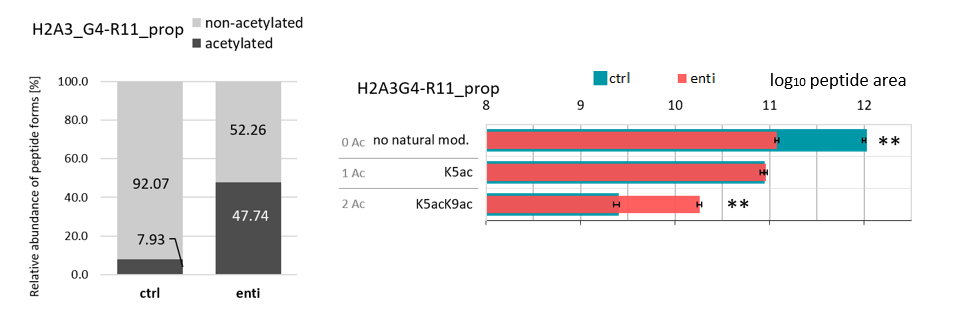


# **Identification parameters of TMA- and Prop-labeled histone peptides**

## **Mass spectral characteristics of TMA-labeled histone peptides**

Inspection of MS/MS spectra of TMA-labeled histones revealed characteristic fragmentation peaks corresponding to masses of lysine immonium (*m/z* 101) and related ions (*m/z* 84 and 112) carrying TMA, including N-terminal lysine fragments carrying TMA together with methylation, dimethylation, and acetylation (see table below). Such ions can be supportive for further unambiguous assignment of a given mass spectrum to a specific post-translationally modified peptide form.

Characteristic low-mass fragment ions observed in MS/MS spectra of TMA-labeled histone peptide forms.

| **Mass of lysine fragment (*m/z*)**  **according to Matrix Science**  <http://www.matrixscience.com/help/fragmentation_help.html> | | 84 | 101 | 112 |
| --- | --- | --- | --- | --- |
| **Modification** | **Monoisotopic mass of modification (*m/z*)** | **Experimental mass of signature ion (*m/z*)** | | |
| **TMA** | 84.058 | **168.138** | **185.164** | **196.133** |
| **TMA+TMA** | 84.058+84.058 |  | **269.222** |  |
| **TMA+me1** | 84.058+14.016 |  | **199.14** | **209.165** |
| **TMA+me2** | 84.058+28.031 |  | **213.197** |  |
| **TMA+ac** | 84.058+42.011 |  | **227.175** |  |

## **Chromatographic behavior of TMA- and Prop-labeled histone peptides**

LC-gradient was optimized with respect to separation of more hydrophobic derivatized peptides. Representative chromatograms of control samples labeled with TMA and Prop are shown to demonstrate the difference in precursor peak distribution across the LC-gradient due to distinct acquired hydrophobicity. The difference in RT of selected peptides is shown in the table below. In case of TMA-labeled samples, peaks at the end of the gradient (RT > 85min) correspond mainly to long peptides containing multiple lysine residues, e.g., N-termini of histones H1 and H2B variants.


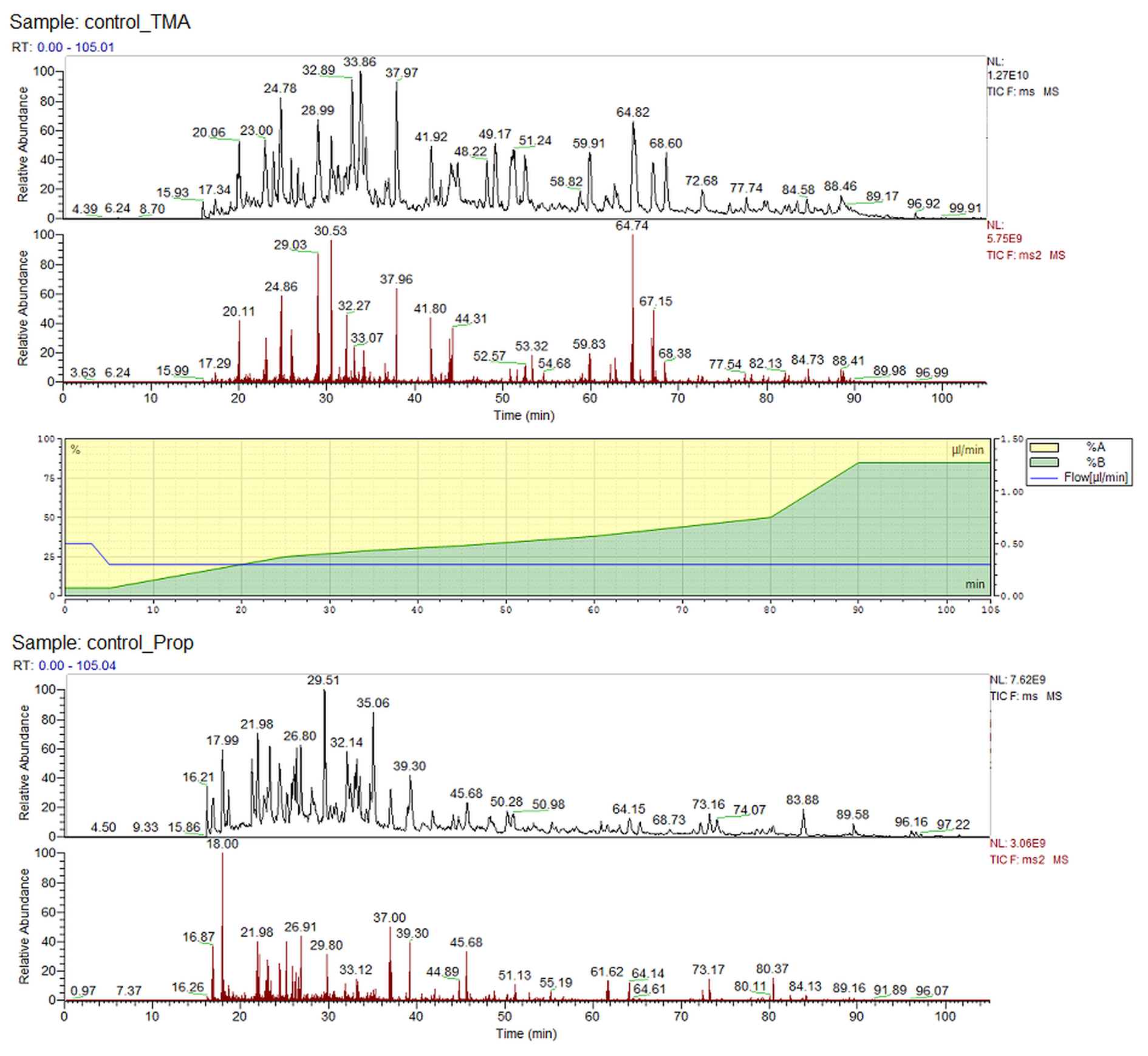


| **RT comparison of selected TMA- and Prop-labeled peptides.** For histones H3 and H4, RT of naturally non-modified and fully post-translationally modified peptide forms after derivatization with TMA and Prop is compared. The impact of derivatization on retention of long peptides with multiple lysine residues is also shown. | | | | | | | |  |
| --- | --- | --- | --- | --- | --- | --- | --- | --- |
| #  (N-term +lysines) | sequence | peptide | **TMA** | | **Prop** | | **∆RT [min]** | |
|  |  |  | *m/z* | RT [min] | *m/z* | RT [min] |  |  |
| 1+4 | **GK**GG**K**GLG**K**GGA**K**R | H4_K5K8K12K16 | 564.3575; 846.0325 | ***69*** | 517.6386; 775.9543 | ***33*** | 36 | |
| 1+0 | **GK**GG**K**GLG**K**GGA**K**R | H4_K5acK8acK12acK16ac | 761.9386 | ***31*** | 747.932 | ***28*** | 3 | |
| 1+2 | **K**STGG**K**APR | H3_K9K14 | 577.3506 | ***43*** | 535.3037 | ***26*** | 17 | |
| 1+0 | **K**STGG**K**APR | H3_K9acK14ac | 535.3037 | ***20*** | 521.2880 | ***17*** | 3 | |
| 1+2 | **K**QLAT**K**AAR | H3_K18K23 | 619.8900 | ***65*** | 577.8482 | ***35*** | 30 | |
| 1+0 | **K**QLAT**K**AAR | H3_K18acK24ac | 577.8482 | ***38*** | 563.8323 | ***32*** | 6 | |
| 1+3 | **K**SAPATGGV**KK**PHR | H3_K27K36K37 | 590.6927; 885.5354 | ***45*** | 553.3177; 829.4728 | ***28*** | 17 | |
|  | | | | | | | |  |
| 1+10 | **P**EPT**K**SAPAP**KK**GS**KK**AVT**K**AQ**KK**DG**KK**R | H2B type 1-D_K5K11K12K15K16K20K23K24K27K28 | 1004.3755 | ***90*** | 1236.0419 | ***48*** | 42 | |
| 1+10 | **P**EPV**K**SAPVP**KK**GS**KK**AIN**K**AQ**KK**DG**KK**R | H2B type 1-M_K5K11K12K15K16K20K23K24K27K28 | 1017.6367 | ***90*** | 940.5522 | ***49*** | 41 | |
| 1+7 | **S**ETAPLAPTIPAPAE**K**TPV**KKK**A**KK**AGATAG**K**R | H1.3_K16K20K21K22K24K25K32 | 986.8431 | ***86*** | 937.7858 | ***56*** | 30 | |

# - number of groups available for labeling

## **Identification of H3K4 in TMA-labeled samples**

Even though increase in hydrophobicity of TMA-labeled peptides allowed to distinguish more modified forms compared to Prop, the detection of H3A1-R8 carrying methylations at K4 remains problematic. In our experiments, only non-modified form of H3A1-R8 was identified and subsequently quantified (*m/z* 550.3327 and RT 25.7 min) by MS-based approach. For comparison between dmso- and enti-samples, raw values of peptide abundance (without normalization) were used. More than 2-times decrease in abundance of non-modified H3K4 was found after enti treatment (with p-value of 0.053 which is slightly above set threshold for significance of difference). The figure below shows the quantitative comparison between dmso and enti, representative EIC exported from Skyline software, and MS/MS spectra with identified fragment ions. No form of H3A1-R8 was detected in Prop-samples. To check the presence of K4-methylated forms in MEC-1 histone extracts, we additionally performed western blots using following antibodies: Anti-Histone H3 monomethyl K4 (ab8895, abcam), Anti-Histone H3 dimethyl K4 (07-030, Merck) and Anti-Histone H3 trimethyl K4 (ab8580, abcam); Anti-Rabbit IgG–Peroxidase antibody (A6154, Merck) was used as secondary antibody and Luminata Crescendo Western HRP substrate (Merck) was used for detection. Representative figures of blots show increased levels of methylated forms in enti-samples compared to controls which correlates with decreased level of non-modified counterparts found by LC-MS/MS. Very low and almost no signal of H3K4me3 was detected in enti and control samples, respectively.


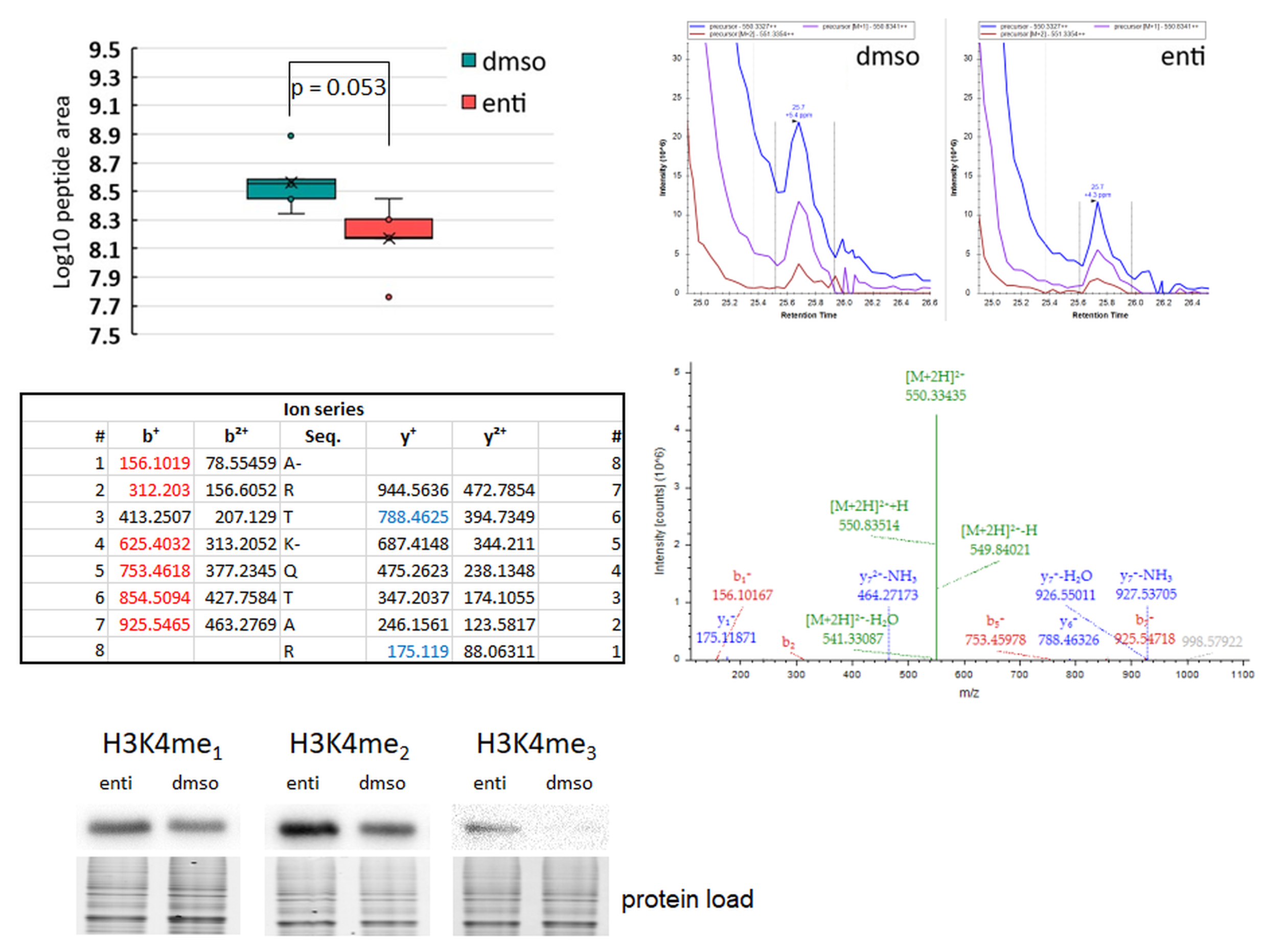


## **Comparison of identified PTMs with the list of annotated PTMs in UniProt database**

The tables below show comparison of identified post-translationally modified sites in TMA- and Prop-samples with PTMs annotated in UniProt database (updated February 2, 2021). Only acetylated and methylated modified forms which were included in our database search are shown. In addition to PTMs annotated in UniProt (black font), several other modified sites were found in TMA-labeled samples (red font). All those sites have been reported in previous studies (7, 8, 22). Ambiguous identifications which are not annotated in UniProt are colored in blue. Several PTMs annotated in UniProt were not detected in our experiments which might reflect the fact that particular PTMs can be related to specific cell types and cultivation conditions.

| **Amino acid modifications of Histone H3.1**  <https://www.uniprot.org/uniprot/P68431#ptm_processing> | | TMA | Prop |
| --- | --- | --- | --- |
| Position(s) | Description; Actions | Site detected | |
| R2 | Asymmetric dimethylarginine; by PRMT6; alternate; 3 Publications | N | N/F |
| K4 | N6,N6,N6-trimethyllysine; alternate; 3 Publications | N | N/F |
|  | N6,N6-dimethyllysine; alternate; 3 Publications | N | N/F |
|  | N6-acetyllysine; alternate; 1 Publication | N | N/F |
|  | N6-methyllysine; alternate; 3 Publications | N | N/F |
| R8 | Symmetric dimethylarginine; by PRMT5; alternate; By similarity | N/F | N/F |
| K9 | N6,N6,N6-trimethyllysine; alternate; 5 Publications | Y | Y |
|  | N6,N6-dimethyllysine; alternate; 5 Publications | Y | Y |
|  | N6-acetyllysine; alternate; 6 Publications | Y | Y |
|  | N6-methyllysine; alternate; 5 Publications | Y | Y |
| K14 | N6-acetyllysine; alternate; 6 Publications | Y | Y |
| R17 | Asymmetric dimethylarginine; by CARM1; alternate; 3 Publications | N | N |
| K18 | N6-acetyllysine; alternate; 3 Publications | Y | Y |
|  | N6-methyllysine; alternate; 2 Publications | N | N |
| K23 | N6-acetyllysine; alternate; 4 Publications | Y | Y |
|  | N6-methyllysine; alternate; 1 Publication | N | N |
| K27 | N6,N6,N6-trimethyllysine; alternate; 4 Publications | Y | Y |
|  | N6,N6-dimethyllysine; alternate; 4 Publications | Y | Y |
|  | N6-acetyllysine; alternate; 2 Publications | Y | Y |
|  | N6-methyllysine; alternate; 4 Publications | Y | Y |
| K36 | N6,N6,N6-trimethyllysine; alternate; 5 Publications | N | Y |
|  | N6,N6-dimethyllysine; alternate; 5 Publications | Y | Y |
|  | N6-acetyllysine; alternate; 2 Publications | Y | Y |
|  | N6-methyllysine; alternate; 5 Publications | Y | Y |
| K37 | N6-methyllysine; 1 Publication | N | N |
|  | N6-acetyllysine; | Y | Y |
| K56 | N6,N6,N6-trimethyllysine; alternate; 2 Publications | N | N |
|  | N6-acetyllysine; alternate; 1 Publication | Y | N |
|  | N6-methyllysine; by EHMT2; alternate; 2 Publications | N | N |
| K64 | N6-methyllysine; alternate; 2 Publications | N/F | N/F |
| K79 | N6,N6,N6-trimethyllysine; alternate; By similarity | N | N |
|  | N6,N6-dimethyllysine; alternate; 4 Publications | Y | Y |
|  | N6-acetyllysine; alternate; 1 Publication | Y | N |
|  | N6-methyllysine; alternate; 4 Publications | Y | Y |
| K115 | N6-acetyllysine; alternate; 1 Publication | N/F | N |
| K122 | N6-acetyllysine; alternate; 2 Publications | Y | N |
|  | N6-methyllysine; alternate; 2 Publications | N | N |

Y – PTM identified in the experiment

N – PTM not identified in the experiment

N/F – corresponding part of amino acid sequence was not covered

PTMs which are not annotated in UniProt are colored in red

| **Amino acid modifications of Histone H4**  <https://www.uniprot.org/uniprot/P62805#ptm_processing> | | TMA | Prop |
| --- | --- | --- | --- |
| Position(s) | Description; Actions | Site detected | |
| S1 | N-acetylserine; 1 Publication | Y | Y |
| R3 | Asymmetric dimethylarginine; by PRMT1; alternate; 3 Publications | N | N |
|  | Omega-N-methylarginine; by PRMT1; alternate; 3 Publications | Y | Y |
|  | Symmetric dimethylarginine; by PRMT5 and PRMT; 7; alternateBy similarity | N | N |
| K5 | N6-acetyllysine; alternate; Combined sources; 2 Publications | Y | Y |
| K8 | N6-acetyllysine; alternate; Combined sources; 2 Publications | Y | Y |
| K12 | N6-acetyllysine; alternate; Combined sources; 3 Publications | Y | Y |
| K16 | N6-acetyllysine; alternate; Combined sources; 3 Publications | Y | Y |
| K20 | N6,N6,N6-trimethyllysine; alternate; 3 Publications | N | N |
|  | N6,N6-dimethyllysine; alternate; 3 Publications | Y | Y |
|  | N6-methyllysine; alternate; 3 Publications | Y | N |
| K31 | N6-acetyllysine; alternate; Combined sources | Y | N |
| K59 | N6-acetyllysine | Y | N |
| K77 | N6-acetyllysine | Y | N |
| K79 | N6-acetyllysine | Y | N |
| K91 | N6-acetyllysine; alternate; 1 Publication | Y | N |

Y – PTM identified in the experiment

N – PTM not identified in the experiment

N/F – corresponding part of amino acid sequence was not covered

PTMs which are not annotated in UniProt are colored in red

| **Amino acid modifications of Histone H2A type 3**  <https://www.uniprot.org/uniprot/Q7L7L0#ptm_processing> | | TMA | Prop |
| --- | --- | --- | --- |
| Position(s) | Description; Actions | Site detected | |
| S1 | N-acetylserine; 1 Publication | Y | Y |
| R3 | Symmetric dimethylarginine; by PRMT5; alternate; By similarity | Y | Y |
| K5 | N6-acetyllysine | Y | Y |
| K9 | N6-acetyllysine | Y | Y |
| K36 | N6-acetyllysine | Y | N |
| K95 | N6-acetyllysine | Y | N |
| K127 | N6-acetyllysine | N | Y |

Y – PTM identified in the experiment

N – PTM not identified in the experiment

PTMs which are not annotated in UniProt are colored in red

| **Amino acid modifications of Histone H2B type 1-L**  <https://www.uniprot.org/uniprot/Q99880#ptm_processing> | | TMA | Prop |
| --- | --- | --- | --- |
| Position(s) | Description; Actions | Site detected | |
| K5 | N6-acetyllysine; alternate; Combined sources; 2 Publications | Y | A |
|  | N6-methylated lysine | N | A |
|  | N6,N6-dimethyllysine | A | A |
|  | N6,N6,N6-trimethyllysine | A | A |
| K11 | N6-acetyllysine; alternate; 1 Publication | Y | Y |
|  | N6-methylated lysine | A | A |
|  | N6,N6-dimethyllysine | A | A |
|  | N6,N6,N6-trimethyllysine | A | A |
| K12 | N6-acetyllysine; alternate; 2 Publications | Y | Y |
|  | N6-methylated lysine | A | N |
|  | N6,N6-dimethyllysine | N | A |
|  | N6,N6,N6-trimethyllysine | A | N |
| K15 | N6-acetyllysine; alternate; 2 Publications | Y | Y |
|  | N6-methylated lysine | A | A |
|  | N6,N6-dimethyllysine | A | A |
|  | N6,N6,N6-trimethyllysine | A | A |
| K16 | N6-acetyllysine; alternate; 1 Publication | Y | Y |
|  | N6-methylated lysine | A | N |
|  | N6,N6,N6-trimethyllysine | A | N |
| K20 | N6-acetyllysine; alternate; 2 Publications | Y | Y |
| K23 | N6-acetyllysine; alternate; By similarity | N | N |
| K46 | N6-methyllysine; alternate; 1 Publication | N/F | N |
| K57 | N6,N6-dimethyllysine; alternate; 1 Publication | N/F | N/F |
| K79 | Dimethylated arginine; By similarity | N | N |
| K85 | N6,N6,N6-trimethyllysine; alternate; By similarity | N | N |
|  | N6-acetyllysine; alternate; By similarity | N | N |
| K108 | N6-methyllysine; alternate; 1 Publication | N | N |
| K116 | N6-methylated lysine; alternate; By similarity | N | N |

Y – PTM identified in the experiment

N – PTM not identified in the experiment

N/F – corresponding part of amino acid sequence was not covered

A – ambiguous identification

Ambiguous identifications which are not annotated in UniProt are colored in blue

## **Sequence coverage and PTMs identified in TMA- and Prop-labeled histone peptides**

Compared to Prop, lower sequence coverage was obtained for histone proteins labeled with TMA. Nevertheless, due to improved chromatographic behavior, higher number of modified peptides including isobaric ones were identified in TMA-labeled samples. More modified forms were identified also within histone H3 and H4 cores and C-terminal tails. Representative figures showing sequence coverage and identified PTMs in histones H3.1, H4, and selected variants of H2A and H2B for TMA and Prop are shown below, reflecting high confident peptides of Rank 1 and Ion Score greater than 30. MS/MS spectra of all modified peptides were manually checked and those with poor fragment ion series were excluded. Identified peptides are colored in blue, modified amino acids in pink, and missing part of sequences in black. Combinatorial pattern identified for particular peptides is depicted in the grids below the respective sequence. Importantly, many of modified peptide forms could not have been subsequently quantified in Prop-labeled samples from MS1-level due to frequent presence of co-eluting peaks.


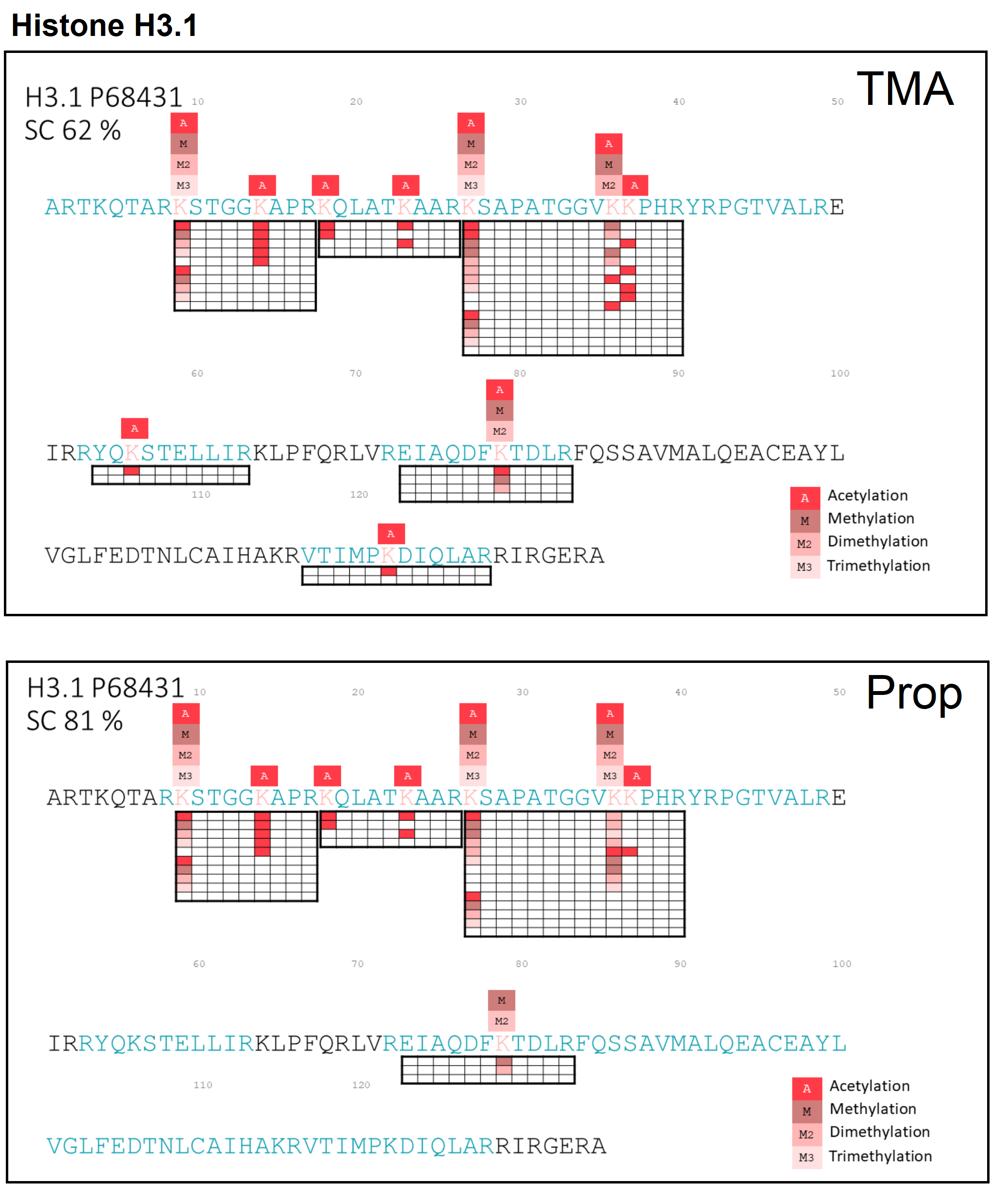


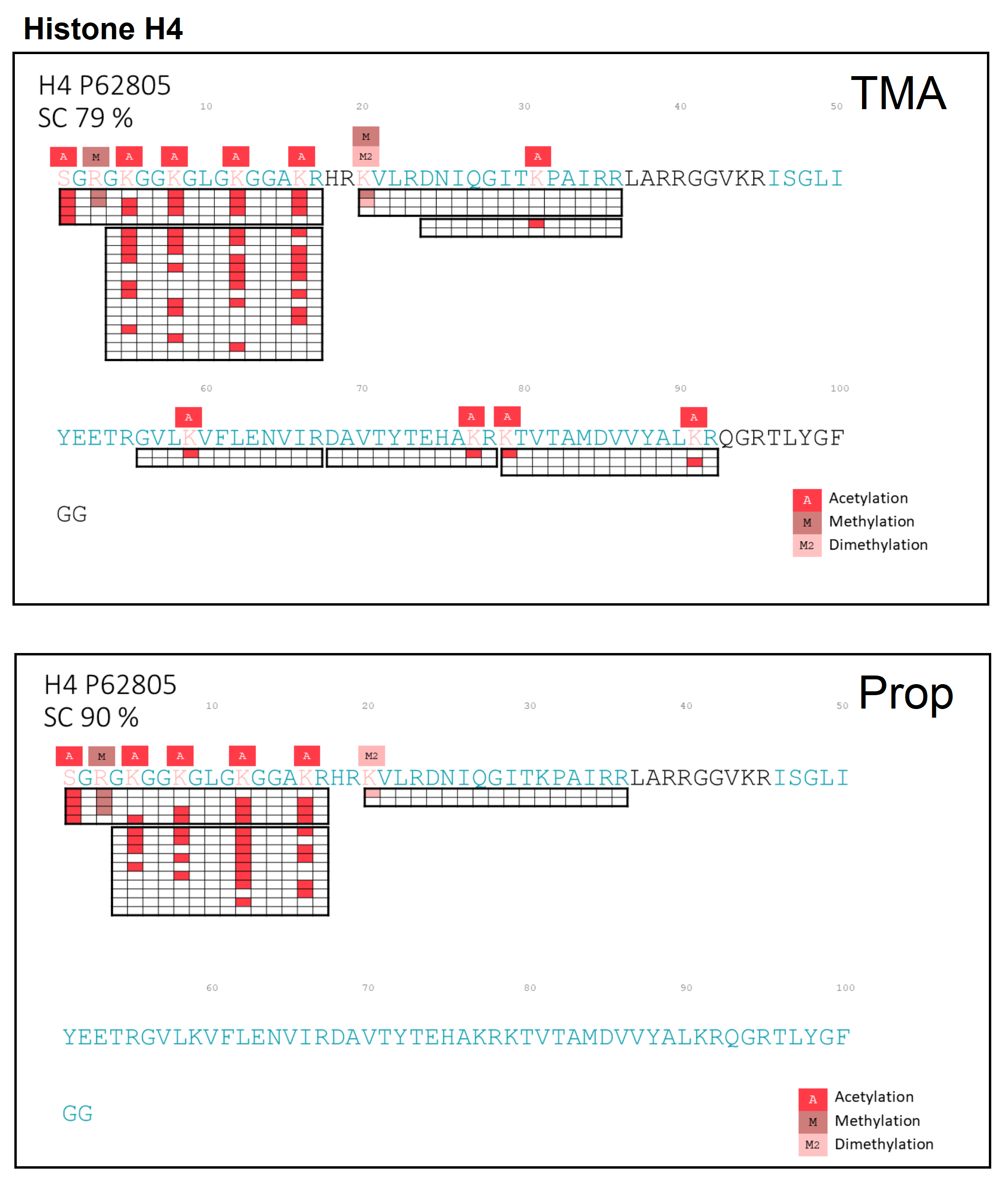


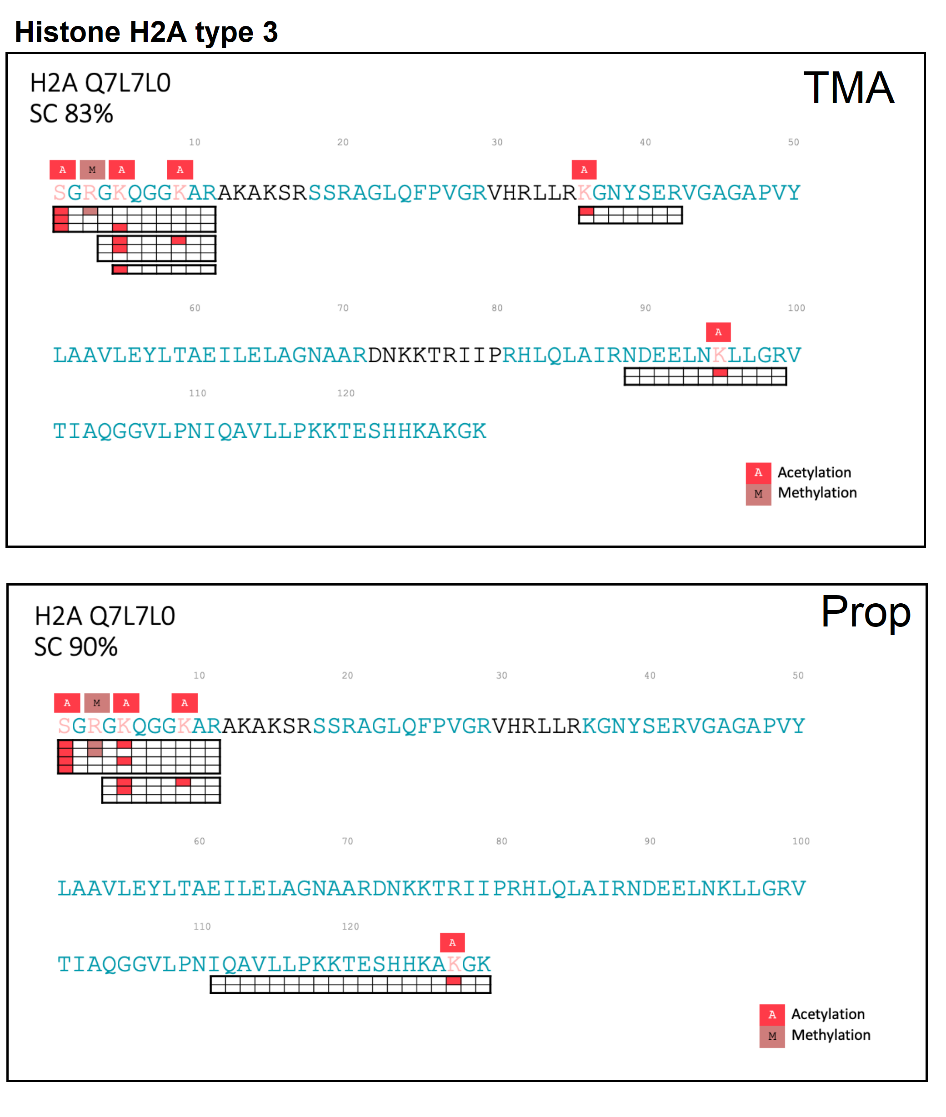


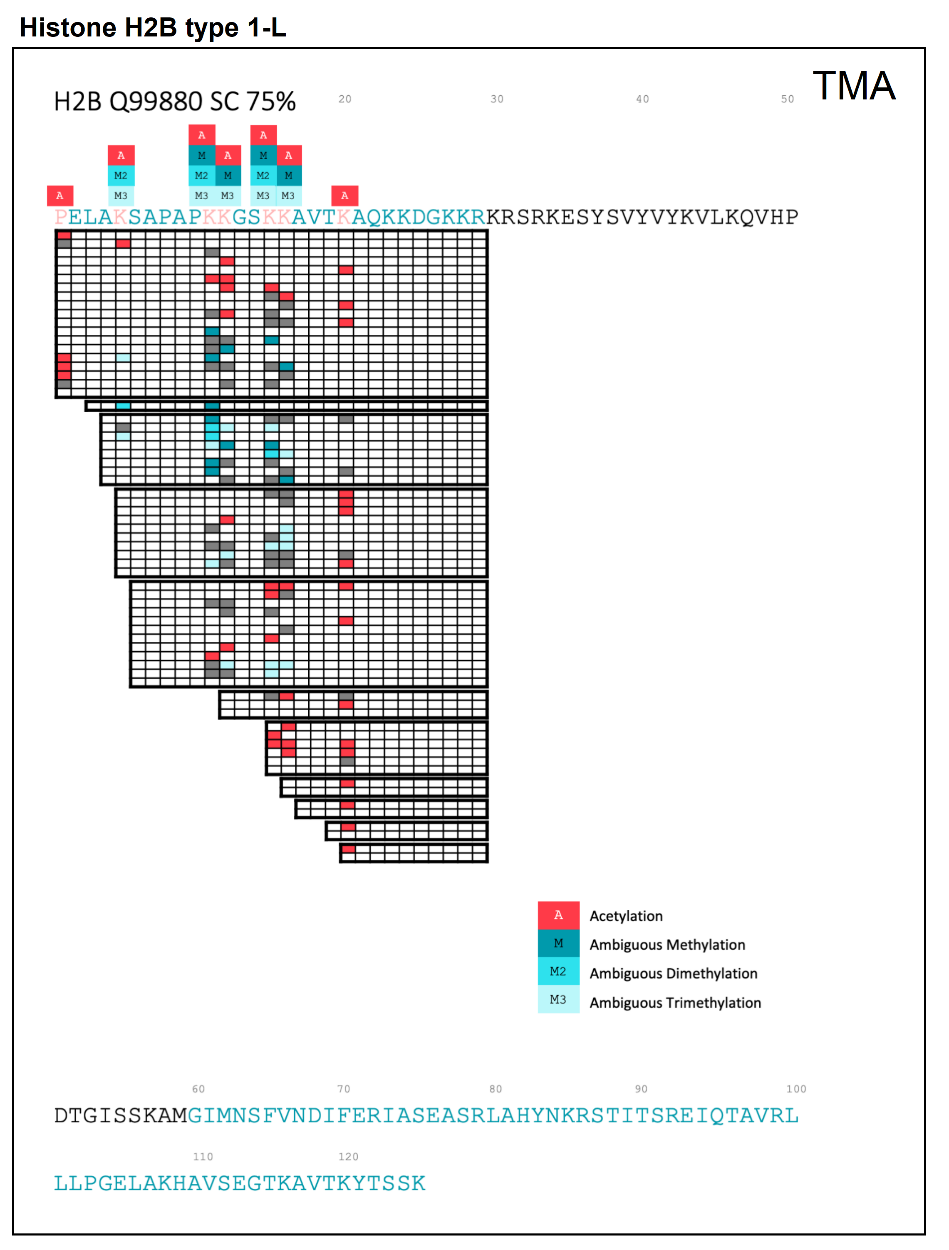


The figure clearly demonstrates the complexity of combinatorial pattern of PTMs in representative H2B variant. Desired tryptic sequence of derivatized N-termini (P1-R29) includes eleven possible sites for modifications, i.e., ten lysine residues and peptide N-terminus. Results from search engine are ambiguous, showing several possible combinations for a single scan. Missing fragment ions in MS/MS spectra make it impossible to determine the exact position and often also the type of modification. In addition, other shorter modified peptide forms („unassignable peptides“) were identified. Even more ambiguous identifications for H2B modified forms were obtained using derivatization with Prop (see the figure below).


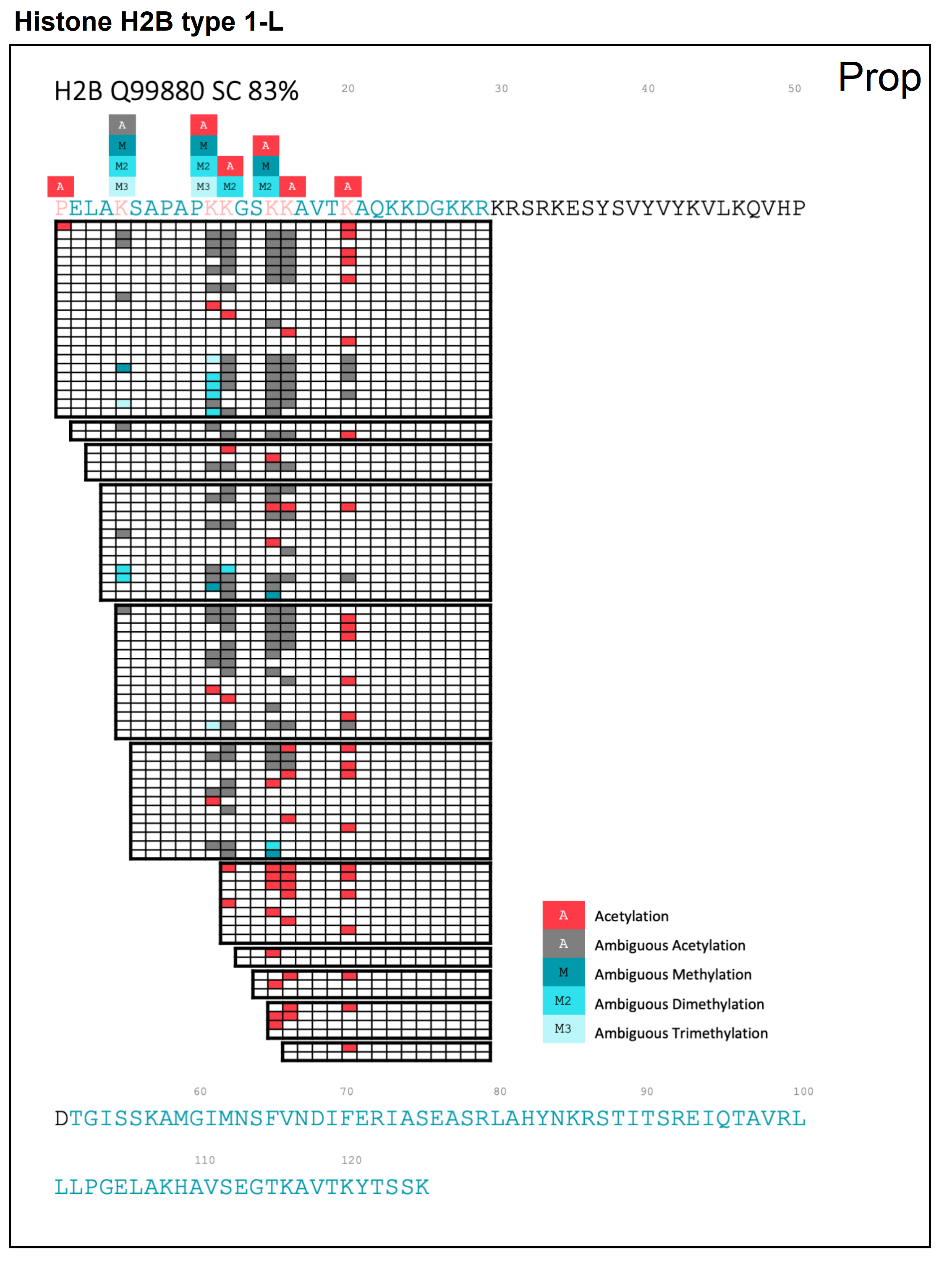


# **References**

7. Zhang, L.,Eugeni, E. E.,Parthun, M. R.,Freitas, M. A. (2003) Identification of Novel Histone Post-Translational Modifications by Peptide Mass Fingerprinting. *Chromosoma* ***112***, 77–86. https://doi.org/10.1007/s00412-003-0244-6.

8. Beck, H. C.,Nielsen, E. C.,Matthiesen, R.,Jensen, L. H.,Sehested, M.,Finn, P.,Grauslund, M.,Hansen, A. M.,Jensen, O. N. (2006) Quantitative Proteomic Analysis of Post-Translational Modifications of Human Histones. *Molecular and Cellular Proteomics* ***5***, 1314–1325. https://doi.org/10.1074/mcp.M600007-MCP200.

16. Sidoli, S.,Bhanu, N. V.,Karch, K. R.,Wang, X.,Garcia, B. A. (2016) Complete Workflow for Analysis of Histone Post-Translational Modifications Using Bottom-up Mass Spectrometry: From Histone Extraction to Data Analysis. *Journal of Visualized Experiments* ***2016***, 1–11. https://doi.org/10.3791/54112.

22. Filippakopoulos, P.,Picaud, S.,Mangos, M.,Keates, T.,Lambert, J. P.,Barsyte-Lovejoy, D.,Felletar, I.,Volkmer, R.,Müller, S.,Pawson, T.,Gingras, A. C.,Arrowsmith, C. H.,Knapp, S. (2012) Histone Recognition and Large-Scale Structural Analysis of the Human Bromodomain Family. *Cell* ***149***, 214–231. https://doi.org/10.1016/j.cell.2012.02.013.
